# Mechanoresponsive ion channels Piezo1 and TRPV4 stimulate ADAM10 and ADAM17 with differential impact on endothelial migration and proliferation

**DOI:** 10.1186/s12964-025-02633-x

**Published:** 2026-01-10

**Authors:** Alessa Pabst, Nathalie Brock, Sofie Hardt, Caroline C. F. Grannemann, Hannah Kubiza, Anja Lena Thiebes, Andreas Ludwig, Aaron Babendreyer

**Affiliations:** 1 Institute of Molecular Pharmacology, University Hospital RWTH Aachen, Aachen, Germany; 2 Department of Biohybrid & Medical Textiles, Institute of Applied Medical Engineering, Helmholtz Institute, RWTH Aachen University, Aachen, Germany

**Keywords:** Mechanotransduction, Mechanosensitive ion channel, Metalloproteinase, Shedding, Adhesion molecules, TNFR1, Shear stress, ADAMs

## Abstract

**Background:**

Endothelial cells are constantly exposed to mechanical forces generated by blood flow regulating endothelial homeostasis. Pathological events such as vascular injury or thrombus formation alter these forces, which are sensed by endothelial cells and can trigger repair mechanisms, including inflammation, proliferation and migration. The mechanical changes can be detected by cells through various mechanosensors, particularly ion channels such as Piezo1 and transient receptor potential vanilloid 4 (TRPV4). One possible pathway for the translation of mechanical signals into cellular responses involves the downstream activation of membrane-bound proteases of the a disintegrin and metalloprotease (ADAM) family. These proteases cleave transmembrane proteins such as growth factors, adhesion molecules and cytokines, thereby regulating inflammation, proliferation and migration. This study aimed to investigate the link between the ion channels Piezo1/TRPV4 and the metalloproteases ADAM10/17.

**Methods:**

Primary human umbilical vein endothelial cells (HUVECs) were treated with pharmacological agonists of Piezo1 and TRPV4 or cultured under flow conditions for mechanical stimulation. Pharmacological inhibitors and shRNA-mediated knockdown were used to assess the involvement of specific proteins. ADAM activity was determined indirectly by measuring the release of substrates such as junctional adhesion molecule A (JAM-A) or tumor necrosis factor receptor 1 (TNFR1) into the culture medium or by a reduction in the cell lysates via ELISA, on the cell surface by flow cytometry, and in the immunocytochemical staining of vascular endothelial cadherin (VE-cadherin). The effects on cell proliferation and migration were analyzed via live-cell microscopy or via proliferation marker genes.

**Results:**

Mechanical stimulation through flow induced the activation of ADAM10 and ADAM17, which was partially mediated by Piezo1 and TRPV4. This connection was confirmed via the use of specific agonists for both ion channels, which have the potential to independently activate ADAM proteases. Furthermore, specifically Piezo1 activation led to ADAM10/17-dependent increases in proliferation and migration.

**Conclusion:**

Piezo1 and TRPV4 contribute to the mechanical activation of ADAM10/17 in endothelial cells and thus may play an important role in regulating endothelial functions. As shown for the regulation of endothelial proliferation and migration by the Piezo1-ADAM axis.

**Graphical Abstract:**

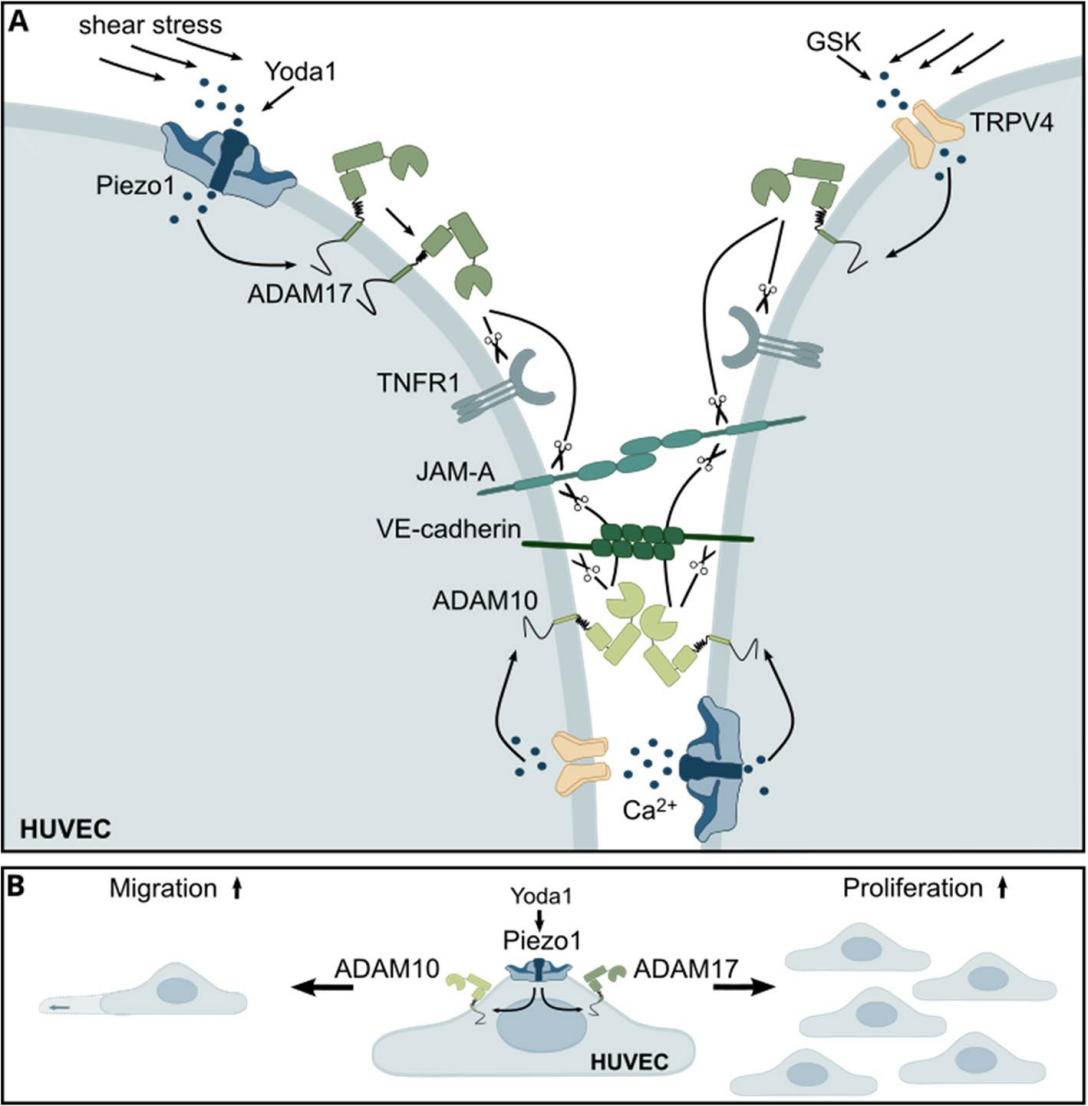

**Supplementary Information:**

The online version contains supplementary material available at 10.1186/s12964-025-02633-x.

## Introduction

Endothelial cells within the cardiovascular system are constantly exposed to changes in fluid shear stress from blood flow. On the one hand, constant laminar flow results in tight cell–cell adhesion, the regulation of proliferation and migration, and an anti-inflammatory phenotype. On the other hand, changes in the magnitude of flow or disturbed flow lead to opposing effects, including vasoconstriction, thrombogenicity, inflammation or cell death. Therefore, sensing these forces is essential for maintaining endothelial integrity and function. Several mechanosensors located on the cell surface are known to be involved in transducing these mechanical forces into cellular responses [1]. A key mechanosensor is the mechanosensitive ion channel Piezo1. The channel is expressed in various cell types and tissues and primarily mediates Ca^2+^ influx upon opening [2]. In the endothelium, Piezo1 is particularly abundant and is suggested to be the dominant mediator of cellular responses to shear stress [3]. Transient receptor potential vanilloid 4 (TRPV4) is a polymodal ion channel that can also be involved in mechanotransduction, resulting in calcium influx upon activation [4]. While Piezo1 is mainly known to be activated by mechanical forces and can also be activated by changes in osmolarity [5], TRPV4 activity can be induced by a variety of stimuli, including changes in osmolarity [6], temperature [7], shear stress [8] and acidity [9]. In mice, Piezo1 appears to be more critical than TRPV4, as *Piezo1* knockout is lethal because of impaired vasculogenesis [10], whereas *Trpv4* knockout is vital [11]. Nevertheless, conditional knockout of both *Piezo1* and *Trpv4* attenuated nitric oxide (NO)-mediated vessel relaxation [12, 13]. Interestingly, a connection between Piezo1 and TRPV4 has been described in endothelial cells, where Piezo1 activation triggers phospholipase A2, subsequently leading to the opening of TRPV4 channels [14]. However, another study reported independent and even contradictory functions in endothelial cells [15]. In particular, Piezo1 is involved in several key endothelial functions, such as cell adhesion, permeability, proliferation and migration [3]. Recently, Piezo1 has also been reported to directly interact with another important shear stress sensor: the mechanosensory adhesion complex containing vascular endothelial cadherin (VE-cadherin), platelet and endothelial cell adhesion molecule 1 (PECAM1 or CD31) and vascular endothelial growth factor receptor 2 (VEGFR2) [16].

Piezo1 and TRPV4 mediate the local influx of cations from the extracellular compartment. Most likely, local changes in Ca^2+^ concentrations are sufficient to induce a number of posttranslational events that regulate protein functions as well as transcriptional changes. For example, shear-induced Piezo1 activation mediates the mRNA transcription of eNOS (endothelial nitric oxide synthase), resulting in vasodilation. Other important effector molecules of shear-induced endothelial responses are adhesion molecules, which contribute to inflammation by controlling vascular permeability and leukocyte transmigration [17–21] or promoting proliferation and migration in terms of vessel repair [22, 23]. Adhesion molecules can be regulated not only at the transcriptional level because of pathological flow patterns or inflammatory stimuli such as intercellular adhesion molecule 1 (ICAM1) and vascular cell adhesion molecule 1 (VCAM1) but also because several endothelial adhesion molecules are regulated at the posttranslational level. These proteins include the tight junction protein junctional adhesion molecule A (JAM-A), the adherens junction proteins VE-cadherin, PECAM1, ICAM1 and VCAM1, which are known substrates of membrane-expressed metalloproteases, including the a disintegrin and metalloprotease (ADAM) family.

ADAM17 and ADAM10 are the most prominent and most investigated members of the ADAM family. They cleave transmembrane proteins on the cell surface, also known as shedding, resulting in the release of the extracellular ectodomain or the induction of intracellular pathways due to the remaining intracellular part. Both ADAM17 and ADAM10 have the potential to cleave various categories of substrates, including cytokines such as tumor necrosis factor α (TNFα), receptors such as tumor necrosis factor receptor 1/2 (TNFR1/2) or Notch1, and growth factors such as heparin binding EGF-like growth factor (HBEGF) or the previously mentioned adhesion proteins. Owing to their broad substrate specificity, ADAMs participate in numerous signaling pathways and contribute to critical processes, especially inflammation and proliferation. One activation mechanism involves the regulation of ADAM10 by Ca^2+^ ionophores or the protein kinase C (PKC)-dependent activation of ADAM17 [24]. This makes them likely downstream targets of mechanically induced Ca^2+^ influx mediated by mechanoresponsive ion channels such as Piezo1 and TRPV4 in endothelial cells. In human microvascular endothelial cells, Piezo1-mediated activation of ADAM10 reportedly leads to the cleavage and subsequent signaling of Notch1 [25]. Recently, we demonstrated that Piezo1 can activate ADAM10 and ADAM17 in lung epithelial cells, resulting in the release of the growth factor amphiregulin and the adhesion molecule JAM-A [26]. However, the regulation of endothelial proteases, especially ADAM17, by Piezo1 and TRPV4 and the functional implications of these processes remain unknown.

In this study, we aimed to address this knowledge gap by investigating the axis between Piezo1 and two members of ADAM proteases for their functional relevance in endothelial cells, as well as the potential of TRPV4 to regulate ADAM activity. Furthermore, we explored whether synergistic or independent effects exist between both ion channels in regulating ADAM activation. The induction of ADAM activity via Piezo1 or TRPV4 was investigated in primary human umbilical vein endothelial cells (HUVECs) by measuring JAM-A and TNFR1 release. The contributions of Piezo1, TRPV4 and ADAMs were verified via the use and combination of specific inhibitors, agonists and lentiviral knockdown. Finally, the functional consequences for the proliferative and migratory behavior of the cells were investigated via live-cell imaging.

## Methods

### Materials

Dimethylsulfoxide (DMSO) was obtained from Roth (Karlsruhe, DE). Phorbol-12-myristat-13-acetat (PMA) was purchased from Biomol (CAY10008014.1; Hamburg, DE). GSK1016790A (GSK) and HC-067047 (HC) were obtained from Selleckchem (Houston, USA). GI254023X (GI) was obtained from GlaxoSmithKline (Brentford, UK). GW280264X (GW), Bisindolylmaleimide II (BIM-2) and Yoda1 were obtained from Tocris (Minneapolis, USA). Salvianolic acid B (SalB), Grammostola mechanotoxin #4 (GsMTx4), KN93, LY294002 (LY), TAE226 (TAE) and SCH772984 (SCH) were purchased from MedChemExpress (Monmouth Junction, USA). U0126 was obtained from Cell Signaling Technology (Massachusetts, USA). For the antibodies used in this study, see the respective method sections. PMA was used as known ADAM17 activator [27] at a concentration of 100 ng/ml to induce ADAM17-mediated shedding in endothelial cells as it was already shown for endothelial cells [28]. GSK and Yoda1 were used as specific TRPV4 and Piezo1 agonists, respectively [5, 29]. GSK was used at a concentration of 0.6 µM and Yoda1 at a concentration of 1 µM. The concentrations were determined as maximal concentration which showed no cytotoxic effect across the different donors (Suppl. Figure 1). To inhibit ADAM10 and ADAM17, we used 10 µM GI254023X (GI; more selective for ADAM10) and 10 µM GW280264X (GW; a dual ADAM10/17 inhibitor). This concentration was shown to efficiently inhibit basal ADAM10- and PMA-induced ADAM17-mediated shedding [30] as well as Yoda1-induced ADAM10 and ADAM17-mediated shedding in lung epithelial cells [26]. HC was used as TRPV4-specific inhibitor at a concentration of 0.3 µM which was shown to significantly inhibit TRPV4 activity [31]. SalB was used as Piezo1 inhibitor at a concentration of 30 µM which was shown to inhibit Piezo1 activity in endothelial cells [32]. GsMTx4 was used as inhibitor for mechanosensitive ion channels [33] at a concentration of 5 µM which was shown to inhibit Piezo1 and migration in endothelial cells [10].

### HUVEC isolation and culture

Human umbilical vein endothelial cells (HUVECs) were isolated from the umbilical cord of cesarean sections in our laboratory, which are covered by the ethics approval EK241/18 from the local ethics committee. For isolation, the vein was incubated with 0.1% collagenase in PBS for 20 min at 37 °C. The dissolved endothelial cells were flushed with PBS, centrifuged at 250 × g for 5 min and washed twice with PBS. The cell pellet was resuspended in Endopan 3 medium (PAN-Biotech, Aidenbach, DE) and seeded into a T25 flask coated with 1% collagen type 1 (Sigma‒Aldrich, St. Louis, USA) in PBS. After 24 h, the remaining erythrocytes were washed out via medium exchange. To rule out contamination with fibroblasts due to prolonged collagenase digestion, the surface expression of the markers CD31 (PECAM-1), CD144 (VE-cadherin) and CD90 (Thy-1) was assessed on the isolated cells by flow cytometry. The cells were exclusively CD31- and CD144-positive and CD90-negative (Suppl. Figure 2), indicating a pure endothelial cell culture.

Wild-type HUVECs were used for experiments until passage 7, and HUVECs stably transduced with shRNA (control shRNA or *PIEZO1*) were used until passage 10. At a confluency of 90%, the cells were used for experiments. The detachment was performed with Accutase (Sigma‒Aldrich, St. Louis, USA) for 4 min. The cells were seeded in well plates 24 h before starting the experiment. The cell concentrations as well as the plate format were adjusted for each method, as described in the respective method section. For the inhibitor treatments, the cells were pre-incubated for 1 h with the inhibitor. After pre-incubation, the inhibitors were also present throughout the entire stimulation experiment. All the treatments were performed with fresh cell culture medium for the indicated time periods.

### Flow experiments

For mechanical stimulation, cells were seeded at a density of 150 000 cells/cm^2^ in 0.2 mm μ-slides (ibidi GmbH, Martinsried, DE), and a 24-well plate was used as a static control. The cells were incubated for 3 h at 37 °C on μ-slides for cell adhesion. To perform specific flow settings, the ibidi pump system (ibidi GmbH, Martinsried, DE) was used to create a laminar flow with a shear stress of 0.4 or 1.5 Pa, which equals 4 or 15 dyne/cm^2^, according to the manufacturer’s protocol. To analyze the released concentration of ADAM substrates in the supernatant, the medium was concentrated from 4 ml to 500 µl (eightfold) using Vivaspins (Sartorius, Göttingen, DE). For mRNA expression analysis, the cells were lysed directly in μ-slides by adding lysis buffer from the RNeasy Kit (Qiagen, Hilden, DE).

### Lentiviral transduction

Lentiviral transduction was performed via short hairpin RNA (shRNA). For lentiviral knockdown, pLKO.1-puro plasmids encoding the shRNA were used. For *PIEZO1* knockdown, the shRNA with the TRC number 0000141714 (sequence: GCTGCTCTGCTACTTCATCAT) and for TRPV4 knockdown, the shRNA with the TRC number 0000045041 (sequence: CTGTTTGACTACGGCACCTAT) were used (Sigma‒Aldrich). The pLKO.1 non-mammalian shRNA plasmid (SHC002) served as a control. Both plasmids contained a sequence for puromycin resistance. HEK293T cells served as the virus producing system. The cells were seeded in a 25 cm dish and co-transfected with 12.5 µg of the specific pLKO.1-puro plasmid, 8.13 µg of psPAX2 and 4.375 µg of pMD2. G (plasmid 12,260 and plasmid 12,259, Addgene, Watertown, USA) using 50 µl of jetPEI® DNA transfection reagent (Polyplus, Illkirch, FR). After 24 h, the medium was exchanged, and after 48 h, the lentivirus-containing supernatant was harvested. To concentrate the lentiviral particles 500 times, ultracentrifugation at 26 000 rpm for 2 h was performed. The pellet was then taken up in 50 µl of PBS and frozen at −80 °C. The transduction of HUVECs was performed by using 2.5 µl virus per 50 000 cells in 2 ml of culture medium supplemented with 5 µg/ml polybrene (Sigma‒Aldrich, St. Louis, USA). Selection was carried out via the use of puromycin dihydrochloride (Sigma‒Aldrich, St. Louis, USA).

### Calcium assay

Intracellular Ca2 + levels were measured via the fluorescent calcium-binding probe Cal-590 AM (AAT Bioquest, Pleasanton, USA). The cells were seeded in a 96-well plate and cultured for 2 days until 100% confluency. The cells were washed once with HEPES buffer, and CAL-590 AM was subsequently added for 2.5 h at 37 °C. After incubation, the cells were carefully washed three times with HEPES buffer and then incubated for 30 min in fresh medium. The calcium trend was measured with a SpectraMax iD3 microplate reader, which automatically injects 50 µl of the stimulants Yoda1, GSK or DMSO diluted in medium in twice the concentration as the final concentration into the wells. The excitation and emission wavelengths were set to Ex./Em. = 540/590 nm. Fluorescence was recorded every 0.5 s for 200 s with a 12.5 s baseline before injection. The baseline mean was then subtracted from the following measurements.

### Enzyme-linked immunosorbent assay

HUVECs were seeded in a 12-well plate at a density of 150 000 cells/well with 1 ml of Endopan 3 and cultured until 100% confluency. After treatment, the supernatant and lysate from the stimulated cells was harvested and cleared from the cell debris by centrifugation (5 min, 16 000 × g, 4 °C). The amounts of soluble JAM-A (JAM-A ELISA kit, SinoBiological, Beijing, China), soluble TNFR1 (human TNFR1 DuoSet ELISA Kit, R&D Systems, Minneapolis, USA) and VE-cadherin in cell lysates (human VE-cadherin DuoSet ELISA Kit, R&D Systems, Minneapolis, USA) were quantified according to the manufacturer’s protocol. The chromogenic reaction substrate BM Blue POD substrate (Roche, Basel, Switzerland) was used for the reaction, which was stopped with 5% H_2_SO_4_. For quantification, a SpectraMax iD3 multimode microplate reader (Molecular Devices, San Jose, USA) was used.

### Flow cytometry

HUVECs were seeded in 6-well plates at a density of 300 000 cells/well and cultured for 24 h until 100% confluency. After treatment, the cells were detached with Accutase (Sigma‒Aldrich, St. Louis, USA) and fixed with 2% PFA for 5 min. The cells were stored or washed with PBS containing 0.2% BSA (flow cytometry buffer). All steps were performed at 4 °C. HUVECs were analyzed for the expression of surface proteins by incubation with a directly labelled or an unlabeled primary antibody for 1 h. If an unlabeled primary antibody was used, cells were washed three times with flow cytometry buffer and subsequently incubated with an APC (allophycocyanin)-conjugated secondary antibody for 45 min. In parallel, corresponding isotype controls were used. The fluorescence signal was detected by a flow cytometer (LRS Fortessa, BD Biosciences, New Jersey, USA) and analyzed with FlowJo10 software (Tree Star, Inc., Ashland, USA). For the final analysis, the geometric mean of the fluorescence signal of the isotype controls was subtracted from the corresponding geometric mean of the respective staining. The following antibodies with the indicated dilutions were used: αJAM-A (1:200, HM-2099, Hycult Biotech), αTNFR1 APC-conjugated (1:20, FAB225A, R&D), αADAM17 (1:50, MAB-9301, R&D), αADAM10 (1:500, MAB-1427, R&D), αVE-cadherin APC REAfinity™ (1:50, 130–125–985, Miltenyi), αCD31 VioBlue REAfinity™ (1:50, 130–110–674, Miltenyi), αCD90 PE REAfinity™ (1:50, 130–114–860, Miltenyi), APC-conjugated goat anti-mouse antibody (1:200, 115–135–164, Jackson ImmunoResearch), IgG1 isotype control (1:100, MAB-002, R&D), IgG2B isotype control (1:500, MAB-004, R&D) REA control antibody REAfinity™ (1:50, APC 130–113–446, PE 130–113–450, VioBlue 130–113–454, Miltenyi).

### Quantitative PCR (qPCR)

The mRNA level was quantified by qPCR and normalized to the mRNA level of the reference gene *RPL13A*. *RPL13A* was used as a reference gene after reference gene analysis via CFX Maestro Software 2.3 (Bio-Rad Hercules, USA). To generate RNA lysates, HUVECs were seeded in a 12-well plate at a density of 150 000 cells/well. After treatment, the cells were lysed, and RNA was extracted via an RNeasy Kit (Qiagen, Hilden, DE). The RNA concentration was quantified photometrically (NanoDrop, Peqlab, Erlangen, DE), and 300 ng of RNA was reverse transcribed with the PrimeScript™ RT Reagent Kit (Takara Bio Europe, St-Germain-en-Laye, FR) according to the manufacturers’ protocol. For the qPCR, a 10 µl volume containing 1 µl cDNA template, 5 µl iTaq Universal SybrGreen Supermix (Bio-Rad, Hercules, USA), 2.9 µl nuclease-free H_2_O and 0.55 µl forward and reverse primer (with 500 nM as final concentration) were used. A list of the primers used and the corresponding annealing temperatures is presented in Suppl. table 9. All the qPCRs were run with the CFX Connect Real-Time PCR Detection System (Bio-Rad, Hercules, USA) via the following protocol: 5 min denaturation at 95 °C, followed by 39 cycles of 10 s denaturation at 95 °C, 10 s annealing at the listed temperatures and 15 s amplification at 72 °C. To determine the efficiency of the uncorrected RFU values, LinRegPCR version 2020.0 software was used. The relative quantification was carried out with CFX Maestro Software 2.3 (Bio-Rad, Hercules, USA).

### Immunocytochemistry and immunofluorescence microscopy analysis

HUVECs were seeded in a 48-well plate at a density of 35 000 cells/well and cultured until 100% confluency. The cells were treated and fixed for 10 min with ice-cold methanol at −20 °C and stored in PBS at 4 °C until staining. The samples were blocked for 30 min at room temperature with 5% (w/v) BSA (Tocris, Minneapolis, USA) in PBS. Primary and secondary antibodies were diluted in 1% BSA (w/v) in PBS and incubated with the fixed cells at 37 °C for 1 h each, with a washing step in PBS between the primary and secondary antibody. A mouse monoclonal VE-cadherin antibody (sc9989, Santa Cruz Biotechnology, Inc., Texas, USA) was used at a concentration of 2 µg/ml. The Alexa Fluor 488-conjugated secondary goat antibody (A11001, Invitrogen AG, Carlsbad, USA) was diluted 1:500. Nuclei were stained with DAPI (D9542, Sigma Aldrich, St. Louis, USA) for 5 min at a concentration of 1.4 mg/ml after permeabilization with Triton X-100. Microscopic analysis was performed with a Leica DMi8 and Leica LAS X software. Images were processed and quantified for integrated fluorescence density with the Integrated Density measurement tool via ImageJ open-source software (National Institutes of Health, Bethesda, MD, USA).

### Live-cell imaging

#### Migration

To investigate changes in migration behavior, 12 000 cells/well were seeded in Imagelock 96-well plates (Sartorius, Göttingen, DE) and cultivated until 100% confluency. An identical wound was simultaneously scratched in the monolayer of all wells via the IncuCyte 96-well Woundmaker Tool (Sartorius, Göttingen, DE). The medium containing detached cells was aspirated, and fresh medium with the appropriate treatments was added. The plates were placed in the IncuCyte SX5 located in an incubator at 37 °C with 5% CO^2^ and 100% humidity until the wound was closed. Images were taken every hour. For quantification, the IncuCyte analysis software (2024A) was used. The relative degree of wound closure over time was calculated and normalized to the wound size at 0 h resulting in a percentage of wound closure presented as relative wound closure.

#### Proliferation

Changes in proliferation behavior were analyzed in 48-well plates with a starting cell number of 5 000 cells per well. The cells were cultured in the IncuCyte SX5 located in an incubator at 37 °C with 5% CO^2^ and 100% humidity until 100% confluency, and images were taken every 4 h. The counted cell numbers were normalized to those at 0 h resulting in a “proliferation index”.

#### Western blot

For Western blotting HUVECs were cultured in 6-well plates at a density of 300 000 cells/well until they reached 100% confluence. After treatment, cells were washed once with PBS and immediately lysed with 500 µl lysis buffer (50 mM Tris; 137 mM NaCl; 2 mM EDTA; 10 mM 1,10-Phenanthroline; pH7.5) supplemented with cOmplete™ protease inhibitor (Sigma-Aldrich, St. Louis, USA) and Marimastat (Sigma-Aldrich, St. Louis, USA). The lysates were cleared by centrifugation (5 min, 16 000 g, 4 °C). Reducing loading buffer (0.1 M Tris–HCl (pH 6.8), 3% (w/v) SDS, 16% glycerol, 8% β-mercaptoethanol, and 0.01% (w/v) bromophenol blue) was added and samples were denaturized 20 min at 60 °C. Proteins were separated by SDS-PAGE and transferred to a polyvinylidene difluoride (PVDF) membrane (Immobilon-FL, Sigma-Aldrich, St. Louis, USA). The membranes were blocked using 1% (w/v) bovine serum albumin (BSA, AppliChem GmbH, Darmstadt, DE) in TBST (50 mM Tris, 150 mM NaCl, 0.1% Tween, pH 7.4) for 20 min at room temperature. Primary antibodies diluted in TBST with 1% (w/v) BSA were incubated overnight at 4 °C. Membranes were washed three times with TBST, followed by incubation with the secondary antibody for 1 h at room temperature. After washing once with TBST and twice with TBS, proteins were detected using the Odyssey 9120 imager system (LI-COR, Lincoln, USA). Afterwards, band intensities were measured with the Image studio Lite software version 5.2 (LI-COR, Lincoln, USA). The following antibodies with indicated dilutions were used: αADAM17 (1:1000, ab39162, Abcam), αGAPDH (1:2000, MA5-15,738, Thermo Scientific), DyLight-680-conjugated αmouse (1:10,000, 35,519, Thermo Scientific), DyLight-800-conjugated αrabbit (1:10,000, 35,571, Thermo Scientific).

#### Data normalization and statistics

Due to the high variability between isolates from different donors, data of the methods mentioned below were normalized using the “Sum of the Replicate” (SoR) method as described by Degasperi et al. [34]. For this, each individual value was divided by the sum of all values within the corresponding data set (one independent experiment). This approach calculates the proportion of each sample’s signal relative to the total signal of the experiment, preserving intra-experimental differences while reducing inter-experimental variability. The data of the following methods were normalized by the SoR normalization procedure: ELISA, flow cytometry, immunofluorescence microscopy, migration, proliferation and western blot. The data of all methods are presented as a “relative” index. The raw values of ELISA and flow cytometry are provided as means in Suppl. Tables 1–8. In contrast, qPCR data was normalized to the mRNA level of the reference gene *RPL13A.*

The quantitative data are presented as the means plus standard deviations (SDs) calculated from a minimum of three independent experiments. Statistics were performed via generalized mixed model analysis (PROC GLIMMIX, SAS 9.4, SAS Institute Inc., Cary, North Carolina, USA) and assumed to have a normal, beta or lognormal distribution with the HUVEC donor conducted as a random effect to assess differences in the size of the treatment effects across the results. Analytical residual plots and the Shapiro‒Wilk test were used to assess homoscedasticity and normal distribution. The degrees of freedom within the model were estimated via the Kenward–Roger approximation. In the case of heteroscedasticity, a nonparametric Kruskal–Wallis test was used. Statistical analysis was performed on values normalized with the SoR method or non-normalized raw data (qPCR). All *p* values were adjusted for multiple comparisons by the false discovery rate (FDR). For immunofluorescence images, the mean values of each donor were determined from ≥ 3 quantified images per condition and then taken as the basis for statistical analysis. For each experiment, at least 3 donors were used. All *p* values < 0.05 were considered significant.

## Results

### Shear stress-mediated activation of ADAM10 and ADAM17 involves Piezo1 and TRPV4 activity

To investigate the role of the mechanosensitive ion channel Piezo1 and the polymodal ion channel TRPV4 in the process of ADAM activation in response to mechanical forces in endothelial cells, we stimulated HUVECs with shear stress to simulate blood flow conditions.

**Fig. 1:**
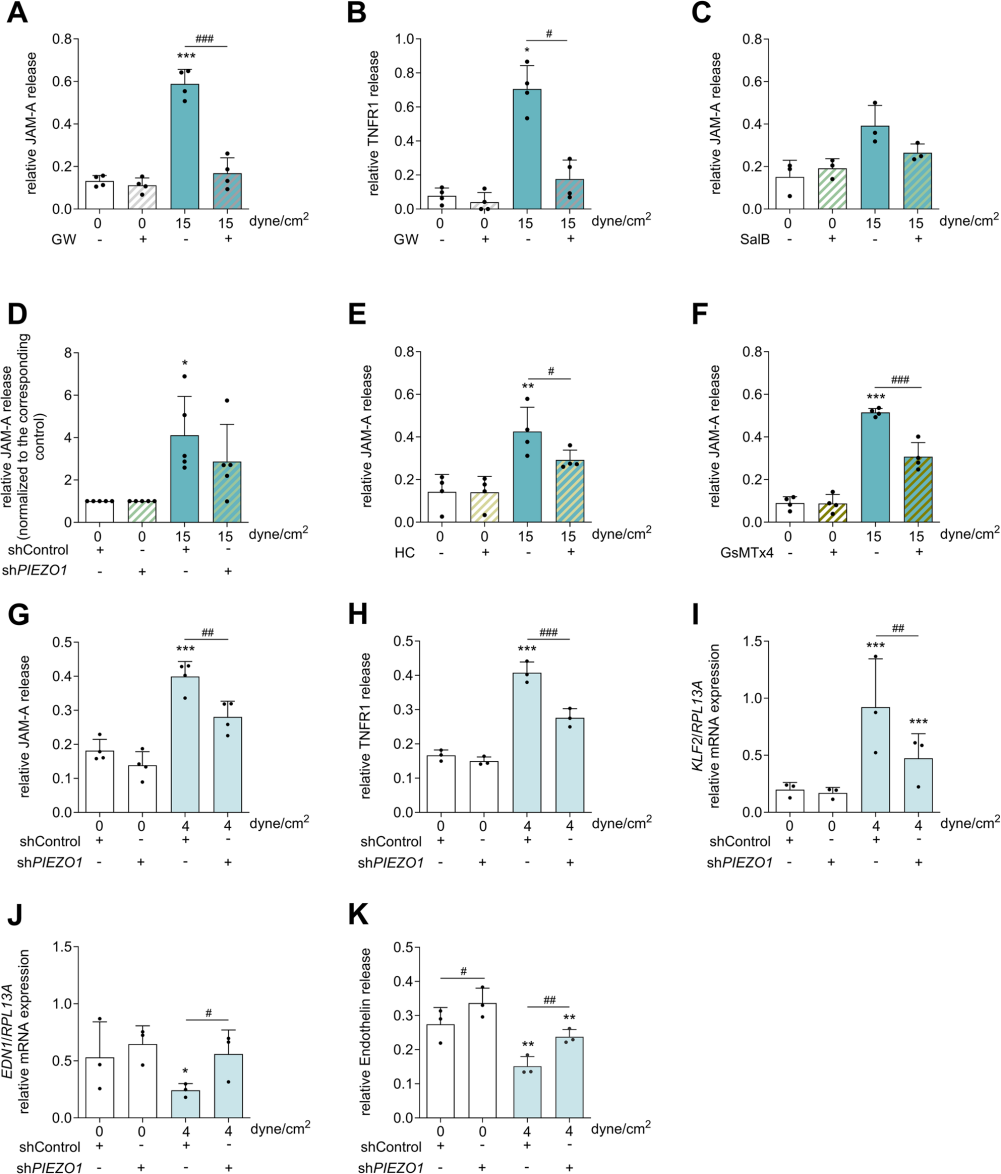
**Flow-induced ADAM activation involves Piezo1 and TRPV4.** HUVECs were treated for 4 h **A**, **B** or 24 h **C**,** D**–**K** with a laminar shear stress of 15 dyne/cm^2^ **A**–**C**,** D**–**F** or 4 dyne/cm^2^ **G**–**K,** and subsequently, the concentrations of soluble JAM-A **A**,** C**,** D**–**G,** soluble TNFR1** B**, **H** or soluble endothelin **K** in the supernatant were measured via ELISA. Furthermore, mRNA expression of *KLF2* or *EDN1* (ET-1) was measured via qPCR **I**,** J**. The involvement of ADAM proteases was studied by adding the ADAM17/10 inhibitor GW (10 µM) **A**, **B**. The role of ion channels was studied by using the Piezo1 inhibitor SalB (30 µM) **C**, the TRPV4 inhibitor HC (0.3 µM) **E**, and the MSC inhibitor GsMTx4 (5 µM) **F**. Additionally, lentiviral transduced shControl or sh*PIEZO1* cells were used to study the effects of *PIEZO1* knockdown under shear stress **D**, **G**–**K**. Quantitative data are shown as the means + SDs of at least three independent experiments. Statistical analysis was performed using a generalized linear mixed model (GLMM) with false discovery rate (FDR) correction as post-hoc test. Statistical differences to the control are indicated by asterisks (* *p* ≤ 0.05, ** *p* ≤ 0.01, *** *p* ≤ 0.001), whereas differences between the treatments are indicated by hashes (# *p* ≤ 0.05, ## *p* ≤ 0.01, ### *p* ≤ 0.001)

HUVECs were exposed for 4 or 24 h in an ibidi perfusion system to flow conditions with a shear stress of 15 dyne/cm^2^ (1.5 Pa). To analyze the shedding activity of ADAM10 and ADAM17, the release of the adhesion molecule JAM-A, a known substrate of both proteases, was measured. Additionally, we studied the release of TNFR1 as an ADAM17-specific substrate. Indeed, stimulation with shear stress led to a significant release of JAM-A and TNFR1 into the supernatant (Fig. 1A, B). To confirm that the increased concentrations of soluble JAM-A and TNFR1 in the endothelial cell supernatant are triggered by the activation of ADAM proteases, we added the small molecule inhibitor GW280264X (GW), which inhibits both ADAM10 and 17 [34]. This resulted in complete suppression of substrate release by GW (Fig. 1A, B), indicating that shear stress promotes ADAM-mediated shedding of endothelial surface molecules.

Next, we investigated the contribution of Piezo1 to the shear stress-induced release of the ADAM substrate JAM-A. To inhibit Piezo1 activity, the Piezo1 inhibitor salvianolic acid B (SalB) [32] was used. However, shear stress-mediated JAM-A shedding was only partially inhibited by SalB (Fig. 1C). To confirm Piezo1 involvement more specifically, *PIEZO1*-knockdown cells were generated by transducing HUVECs with a lentivirus encoding a shRNA against *PIEZO1*. As a control, untargeted control shRNA was used. The efficiency of the knockdown was assessed at the mRNA expression level via qPCR. The knockdown efficiency varied between 54 and 85% (Suppl. Figure 3 A). Similar to the chemical inhibitor, the knockdown of *PIEZO1* had the same inhibitory effect on shear stress-induced shedding (Fig. 1D). Next, we studied the contribution of TRPV4 to shear stress-induced substrate shedding via the specific small-molecule antagonist HC-067047 (HC). The treatment led to a partial reduction in JAM-A shedding (Fig. 1E). To further validate the general involvement of other mechanosensitive ion channels in addition to Piezo1 and TRPV4, the inhibitory spider venom peptide Grammostola mechanotoxin #4 (GsMTx4) was used, which is well known to block Piezo1 but can also inhibit the activation of other ion channels, including TRPV4 [35–38]. Interestingly, GsMTx4 reduced the release of the ADAM substrate JAM-A more profoundly than SalB or HC did but also not completely (Fig. 1F). These findings indicate the involvement of both Piezo1 and TRPV4 in activating ADAM proteases under shear stress. However, since the inhibition was not complete, mechanosensors other than ion channels may also be involved in this response.

To exclude the possibility that the observed effects are specific for physiologically high shear stress, the experiments were additionally performed with a pathophysiologically low shear stress of 4 dyne/cm^2^ (0.4 Pa) for 24 h with *PIEZO1*-knockdown cells. Low shear stress also induced increased release of ADAM substrates, but again, *PIEZO1* knockdown led to only partial inhibition of JAM-A and TNFR1 release (Fig. 1G, H). We also controlled two typical marker genes for endothelial responsiveness to shear stress, *EDN1* (endothelin-1) and *KLF2* (krüppel-like factor 2). Both *EDN1* downregulation and *KLF2* induction were reduced but not completely abolished by *PIEZO1* knockdown (Fig. 1I-K).

Taken together, these data suggest that mechanical stimulation with shear stress strongly activates ADAM10 and ADAM17. This response is at least partially but most likely not exclusively mediated by the ion channels Piezo1 and TRPV4 in endothelial cells.

### Chemical Piezo1 and TRPV4 activation promotes the release of ADAM10- and ADAM17-specific substrates in endothelial cells

**Fig. 2:**
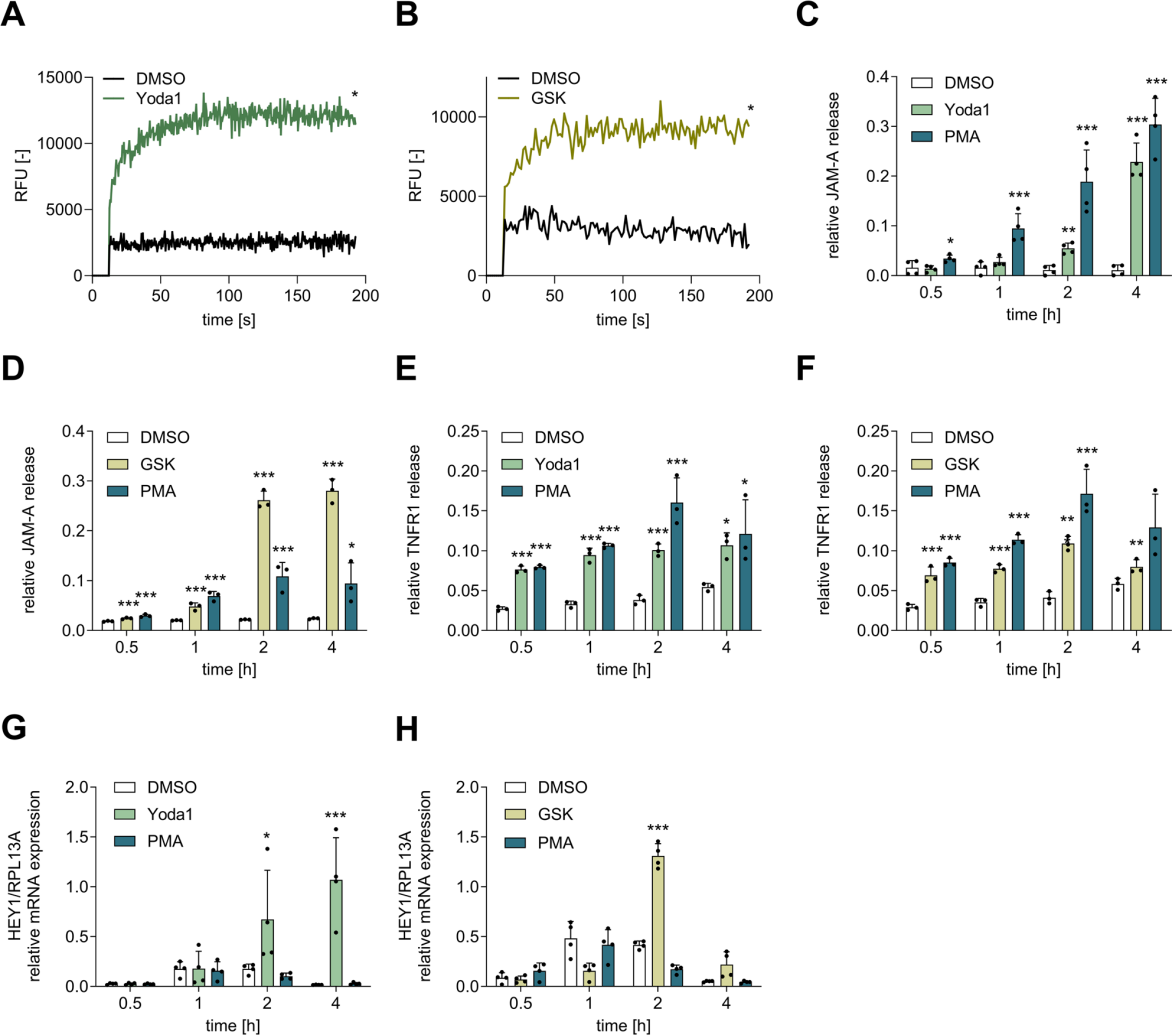
**Release of ADAM10- and ADAM17-specific substrates is induced by chemical activation of Piezo1 and TRPV4.** HUVECs incubated with the fluorescent probe Cal-590 AM (5 µM) were treated with 1 µM Yoda1 or 0.6 nM GSK and Ca2 + influx was measured immediately over a 2 min period with measurements every 0.5 s **A**, **B**. For release measurements, the cells were treated with 1 µM Yoda1, 0.6 nM GSK or 100 nM PMA, as a positive control for ADAM17 activation, for 0.5, 1, 2, or 4 h. The concentrations of soluble JAM-A **C**, **D** or soluble TNFR1 **E**, **F** were measured in the supernatant. Additionally, *HEY1* mRNA expression was measured in relation to the expression of the reference gene *RPL13A* in the cell lysates. **G**, **H**. Quantitative data are shown as the means + SDs of at least three independent experiments. Statistical analysis was performed using a generalized linear mixed model (GLMM) with false discovery rate (FDR) correction as post-hoc test. Statistical differences to the control are indicated by asterisks (* *p* ≤ 0.05, ** *p* ≤ 0.01, *** *p* ≤ 0.001), whereas differences between the treatments are indicated by hashes (# *p* ≤ 0.05, ## *p* ≤ 0.01, ### *p* ≤ 0.001)

To further validate and investigate the Piezo1/TRPV4–ADAM axis mentioned above, we used the well-described chemical agonist Yoda1 to specifically activate Piezo1 [5] and GSK1016790A (GSK) to specifically activate TRPV4 [29] in HUVECs (Fig. 2). As a reference, we included the PKC activator phorbol-12-myristate-13-acetate (PMA), which is a well-established inducer of ADAM17 activity [27]. In the first step, the ability of these agonists to induce a response was tested via Ca^2+^ measurements. Under the chosen conditions, a strong increase in intracellular Ca^2+^ was observed (Fig. 2A, B). To analyze the effects on ADAM10- and ADAM17-mediated shedding, the release of the adhesion molecule JAM-A as combined ADAM10 and ADAM17 substrate was determined. First, we performed time kinetics to determine adequate conditions for further experiments. For JAM-A, a time-dependent increase in JAM-A release was detected after stimulation of Piezo1 and TRPV4 with Yoda1 or GSK, respectively (Fig. 2C, D). The increased release of JAM-A became significant starting from 2 h of treatment with Yoda1 and 1 h of treatment with GSK. The greatest differences compared with those of the control were observed after 4 h of treatment. As specific ADAM17 substrate we also studied TNFR1 release. Induction of TNFR1 shedding was already observed after 0.5 h of stimulation with either Yoda1 or GSK. Thus, TNFR1 shedding occurs more rapidly than JAM-A shedding does (Fig. 2E, F). Of note, no transcriptional changes were observed for either JAM-A (*F11R*) or TNFR1 (*TNFRSF1A*) in response to Yoda1 or GSK treatment at relevant time points, which indicates that the increased release was not due to transcriptional regulation (Suppl. Figure 3B-E). In line with increased JAM-A and TNFR1 release in response to Yoda1, we also observed reduced surface levels of these proteins after 0.5 h Yoda1 treatment. However, this reduction was not visible anymore after 4 h (Suppl. Figure 4A-D). To assess ADAM10-specific shedding activity by a preferred ADAM10 substrate, we investigated the mRNA expression levels of the Notch1 target gene *HEY1* (Hairy/enhancer-of-split related with YRPW motif protein 1)*,* which is an indirect indicator of ADAM10-mediated Notch1 shedding. In line with the increased release of JAM-A and TNFR1, we also observed increased mRNA expression of *HEY1* (Fig. 2G, H) compared to the negative control (DMSO) after 2 h of GSK and 4 h of Yoda1 stimulation.

Interestingly, PMA stimulation resulted in a stronger shedding of JAM-A compared to Yoda1 (Fig. 2C), and generally in a slightly higher shedding of TNFR1 as well (Fig. 2E and F). However, in comparison to GSK-induced JAM-A shedding, the effect was considerably weaker (Fig. 2D). Furthermore, PMA had almost no effect on the induction of the Notch target gene *HEY1*, especially when compared to Yoda1 and GSK (Fig. 2G and H). This confirms the specific activation of ADAM17 by PMA and additionally suggests that Yoda1- and GSK-induced ADAM activation is not, or only partially, mediated through PKC activation, in contrast to the PMA-induced pathway.

These data are in agreement with a previous report [25] that chemical activation of Piezo1 promotes increased mRNA expression of a Notch1 target gene. Additionally, Piezo1 activation strongly promotes the release of JAM-A and TNFR1, which suggests increased ADAM17 activity. Moreover, the mechanoresponsive ion channel TRPV4 has the same ability to induce the release of ADAM-specific substrates as Piezo1 does.

### Cleavage and release of substrates upon specific chemical activation of Piezo1 and TRPV4 is specifically mediated by ADAM10/17

**Fig. 3:**
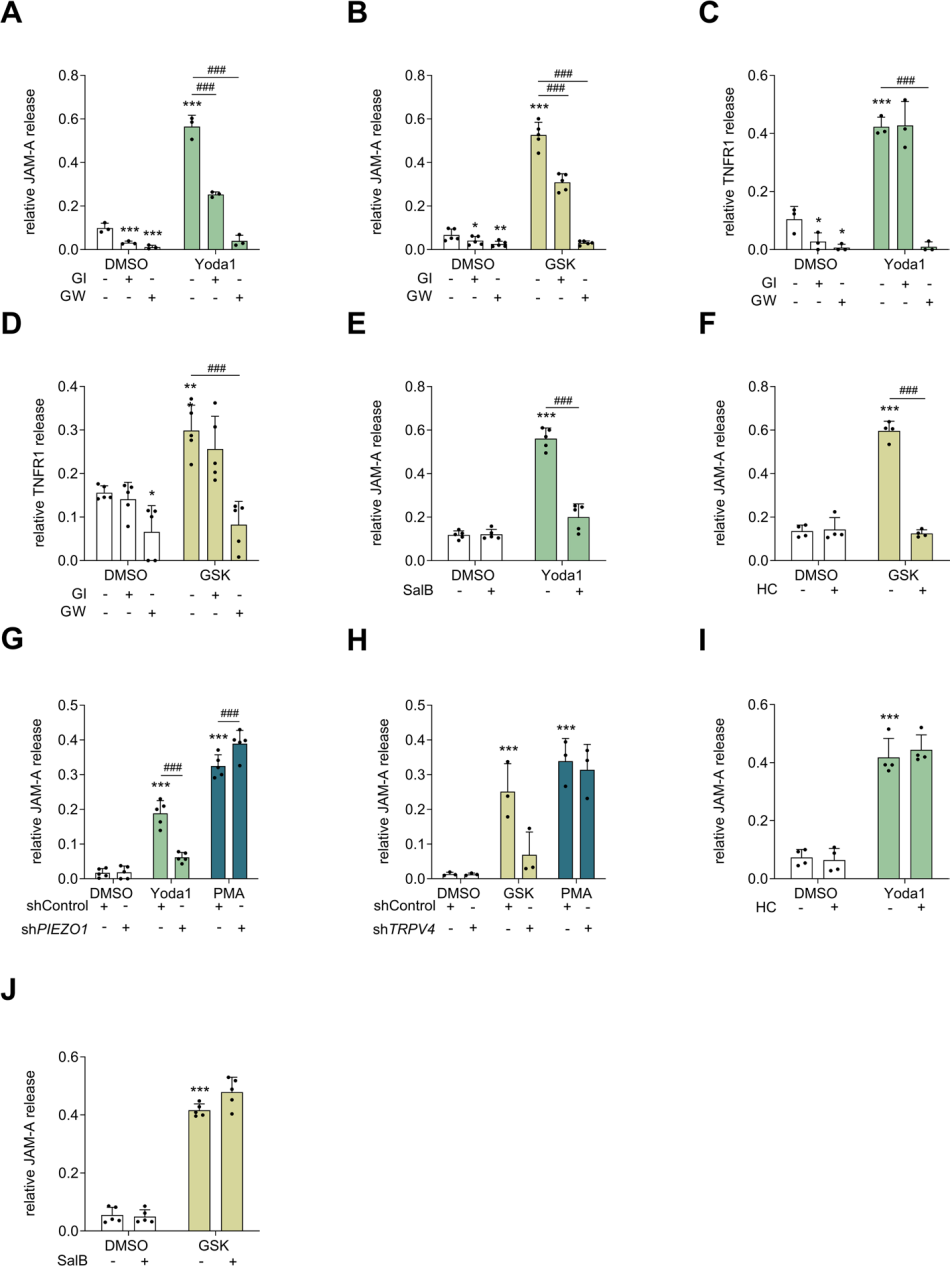
**Ion channel-mediated JAM-A and TNFR1 release is specific for the corresponding mechanoresponsive ion channel and ADAM protease.** HUVECs were pre-incubated with either the ADAM10 inhibitor GI (10 µM), the ADAM17/10 inhibitor GW (10 µM) **A**–**D**, the Piezo1 inhibitor SalB (30 µM) **E, J**, or the TRPV4 inhibitor HC (0.3 µM) **F, I** for 1 h, followed by the addition of Yoda1 (1 µM) **A, C, E, I** or GSK (0.6 nM) **B, D, F, J** and further incubation for 4 h. HUVECs lentivirally transduced with shControl, sh*PIEZO1* or sh*TRPV4* were treated with Yoda1 (1 µM) **G**, GSK (0.6 nM) **H** or PMA (100 nM) as a positive control for ADAM17 activation **G**, **H** for 4 h. Concentrations of soluble JAM-A **A**, **B** and **E**–**J**, or TNFR1 **C**,** D**, were determined via ELISA. Quantitative data are shown as the means + SDs of at least three independent experiments. Statistical analysis was performed using a generalized linear mixed model (GLMM) with false discovery rate (FDR) correction as post-hoc test. Statistical differences to the control are indicated by asterisks (* *p* ≤ 0.05, ** *p* ≤ 0.01, *** *p* ≤ 0.001), whereas differences between the treatments are indicated by hashes (# *p* ≤ 0.05, ## *p* ≤ 0.01, ### *p* ≤ 0.001)

To further investigate whether the induction of JAM-A and TNFR1 release is mediated by ADAM10, ADAM17 or both, the inhibitor GI254023X (GI), which blocks ADAM10 100-fold more potently than ADAM17, was compared to GW, which blocks ADAM10 and ADAM17 with equal potency [34]. To ensure complete inhibition, GI and GW were added 1 h prior to Yoda1 or GSK stimulation. JAM-A release after stimulation with Yoda1 or GSK was significantly decreased by the addition of the ADAM10 inhibitor GI and was completely blocked by the addition of the ADAM10/17 inhibitor GW (Fig. 3A, B). In contrast, the increase in TNFR1 release after Yoda1 or GSK stimulation was not affected by GI but was also entirely prevented by GW (Fig. 3C, D). These results indicate that not only ADAM10 but also ADAM17 can be activated via both ion channels. Therefore, mechanically induced shedding is not limited to ADAM10 substrates but also includes ADAM17 substrates on endothelial cells.

The induction of ADAM-mediated substrate shedding by the chemical agonists Yoda1 and GSK also allowed us to control whether the inhibitors SalB (Piezo1) and HC (TRPV4) effectively block Piezo1 and TRPV4, respectively, under the chosen conditions. The cells were pretreated with the inhibitors 1 h before treatment. Both inhibitors prevented strong increases in ADAM10 and ADAM17 activity (Fig. 3E, F). We also confirmed this result with the knockdown of *PIEZO1* and *TRPV4*, which prevented the induction of ADAM activity in response to Yoda1 and GSK (Fig. 3G, H) as well, although the measured knockdown efficiency was only 58–69% on the transcriptional level (Suppl. Figure 3 A, 6 A).

Since Piezo1 was previously reported to be an upstream regulator of TRPV4, we also investigated whether the activity of these two channels depends on each other. This finding helped us to exclude the possibility that ADAM activation requires both channels working together. To address this, Piezo1 inhibition was combined with TRPV4 stimulation, and TRPV4 inhibition was combined with Piezo1 stimulation. In both cases, the inhibitors had no effect (Fig. 3I, J). These findings suggest that ADAM activity can be induced independently by Piezo1 or TRPV4 activation.

To gain more insight into the regulatory mechanism of ion channel-induced ADAM activation we also examined a possible transcriptional and surface regulation of ADAM10 and ADAM17 in response to Yoda1 or GSK over time. The mRNA expression analysis of ADAM10 and ADAM17 indicated only minor transcriptional changes (Suppl. Figure 5A-D). Also, the surface expression was not affected, besides a slight downregulation of ADAM17 after 0.5 h Yoda1 or GSK stimulation (Suppl. Figure 5E-F). Interestingly, Piezo1 activation induced a slight transient increase of pro and mature ADAM17 in the cell lysates within 0.5 h (Suppl. Figure 5I, J, K). However, overall, no strong transcriptional and translational regulation of ADAMs in response to Yoda1 and GSK could be observed suggesting that the active state of ADAMs is directly affected, which is usually accompanied by a conformational change on the surface that facilitates substrate accessibility.

Interestingly, PMA treatment strongly increases *ADAM17* mRNA expression over time, whereas Yoda1 or GSK stimulation had only slight or no effect (Suppl. Figure 5C-D). To further validate that there is a different mechanism between PMA-induced and Piezo1/TRPV4-induced ADAM activation, we additionally investigated the involvement of PKC in the process of ADAM activation after ion channel stimulation and PMA treatment. As expected, the shedding of JAM-A or TNFR1 induced by Yoda1 or GSK stimulation was not affected by the pan-specific PKC inhibitor BIM-2 (bisindolylmaleimide II), whereas PMA-induced shedding, as a control, was strongly inhibited (Suppl. Figure 6B-D). This finding is in line with our previous findings in lung epithelial cells [26], confirming an independent mechanism of the ion channel-induced ADAM activity from the PKC-mediated pathway.

In this context, we tried to further unravel the link between Piezo1/TRPV4 and ADAM10/17 activation and hypothesized that it may involve phosphorylation cascades. We tested several inhibitors targeting kinases known to be activated downstream of Piezo1/TRPV4 stimulation and/or implicated in ADAM regulation. The inhibition of calcium/calmodulin-dependent proteinkinase II (CaMKII) and focal adhesion kinase (FAK) indicated no participation of these kinases in the ADAM activation process. Whereas interestingly, the inhibition of mitogen-activated protein kinase 1/2 (MEK1/2), extracellular-signal regulated kinase 1/2 (ERK1/2) and phosphoinositide-3-kinase (PI3K) slightly reduced the Yoda1-mediated increase in JAM-A release. Overall, this reflects the complexity of the activation mechanism and indicates that not only one kinase participate in the regulation of the Piezo1-ADAM axis, but a combination of various signaling pathways is likely (Suppl. Figure 7).

### Chemical Piezo1 activation induces ADAM-dependent loss reduction of VE-cadherin

**Fig. 4:**
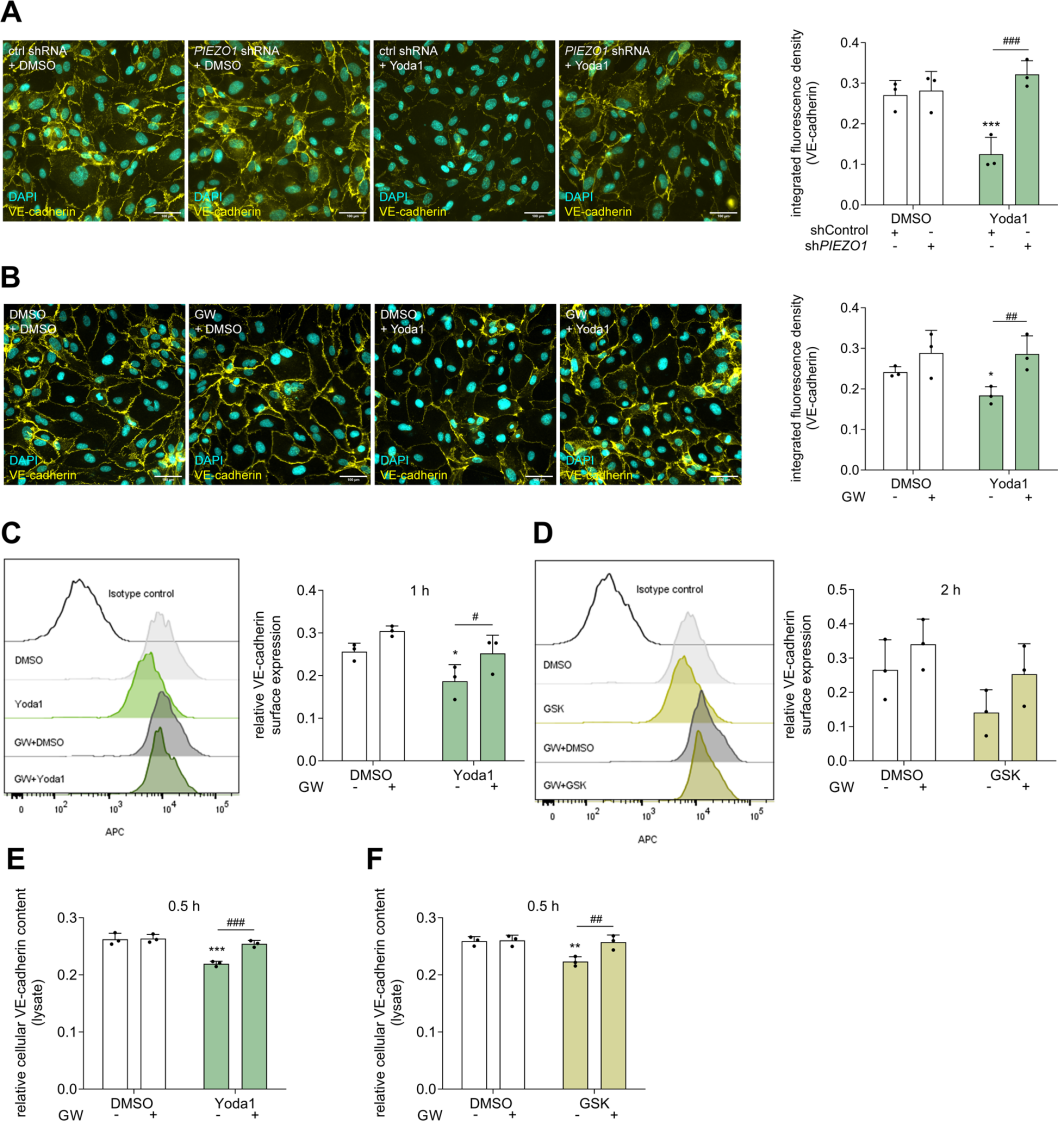
**Piezo1 mediates the ADAM-dependent loss of VE-cadherin.** Lentivirally transduced HUVECs with control shRNA or *PIEZO1* shRNA were stimulated with either DMSO or Yoda1 (1 µM) for 4 h **A**. Non-transduced HUVECs were pre-incubated with DMSO or GW (10 µM) for 1 h, followed by stimulation with DMSO, Yoda1 (1 µM) for 0.5 h **B**, **E** or 1 h **C**, or GSK (0.6 nM) for 2 h **D**, or 0.5 h** F**. For immuncytochemistry **A**, **B** and flow cytometry **C**, **D**, the cells were fixed subsequently and stained for VE-cadherin. For ELISA analysis, the cells were lysated after stimulation and the remaining cellular content was quantified. The integrated fluorescence density was calculated as the mean of at least three images per donor for three independent donors. The quantitative data are shown as the means + SDs. Statistical analysis was performed using a generalized linear mixed model (GLMM) with false discovery rate (FDR) correction as post-hoc test. Statistical differences to the control are indicated by asterisks (* *p* ≤ 0.05, ** *p* ≤ 0.01, *** *p* ≤ 0.001), whereas differences between the treatments are indicated by hashes (# *p* ≤ 0.05, ## *p* ≤ 0.01, ### *p* ≤ 0.001)

It is possible that ADAM10/ADAM17-dependent shedding and release of adhesion molecules after Piezo1 activation contribute to the remodeling of cell junctions. As a cell junction protein crucial for endothelial barrier function, we examined the cellular localization and the cleavage of VE-cadherin. Cells with and without *PIEZO1* knockdown were stimulated with Yoda1 for 4 h and stained for VE-cadherin. We observed a significant reduction in the integrated fluorescence density of VE-cadherin in the cells expressing control shRNA after Yoda1 treatment. This effect was abolished in cells expressing *PIEZO1* shRNA (Fig. 4A). To study the involvement of ADAM10 and ADAM17 in this response, HUVECs were treated with the ADAM10 and ADAM17 inhibitor GW before stimulation with Yoda1. This prevented the Yoda1-induced reduction in VE-cadherin density described above for *PIEZO1* knockdown (Fig. 4B). Additionally, flow cytometric analysis revealed a significant decrease in VE-cadherin surface levels already after 1 h Piezo1 stimulation (Fig. 4C), whereas activation of TRPV4 with GSK induced this decrease more slowly, with the strongest effect after 2 h (Fig. 4D). Furthermore, cell lysates were analyzed for total VE-cadherin expression via ELISA. The previously observed reduction on the surface expression was also reflected by a diminishment in total cellular VE-cadherin content in the lysate after Piezo1 and TRPV4 activation (Fig. 4E, F). Both the reduced surface expression and the decrease in total cellular VE-cadherin content could be attenuated by GW confirming an ADAM-dependent reduction. Taken together, these data suggest that Piezo1 activation leads to reduced VE-cadherin expression at cell junctions via ADAM protease activity. Notably, as observed for JAM-A and TNFR1 (Suppl. Figure 3B-E), also VE-cadherin is not influenced on the transcriptional level by Yoda1 or GSK stimulation (Suppl. Figure 8 A, B).

### Piezo1 affects endothelial migration and proliferation

**Fig. 5:**
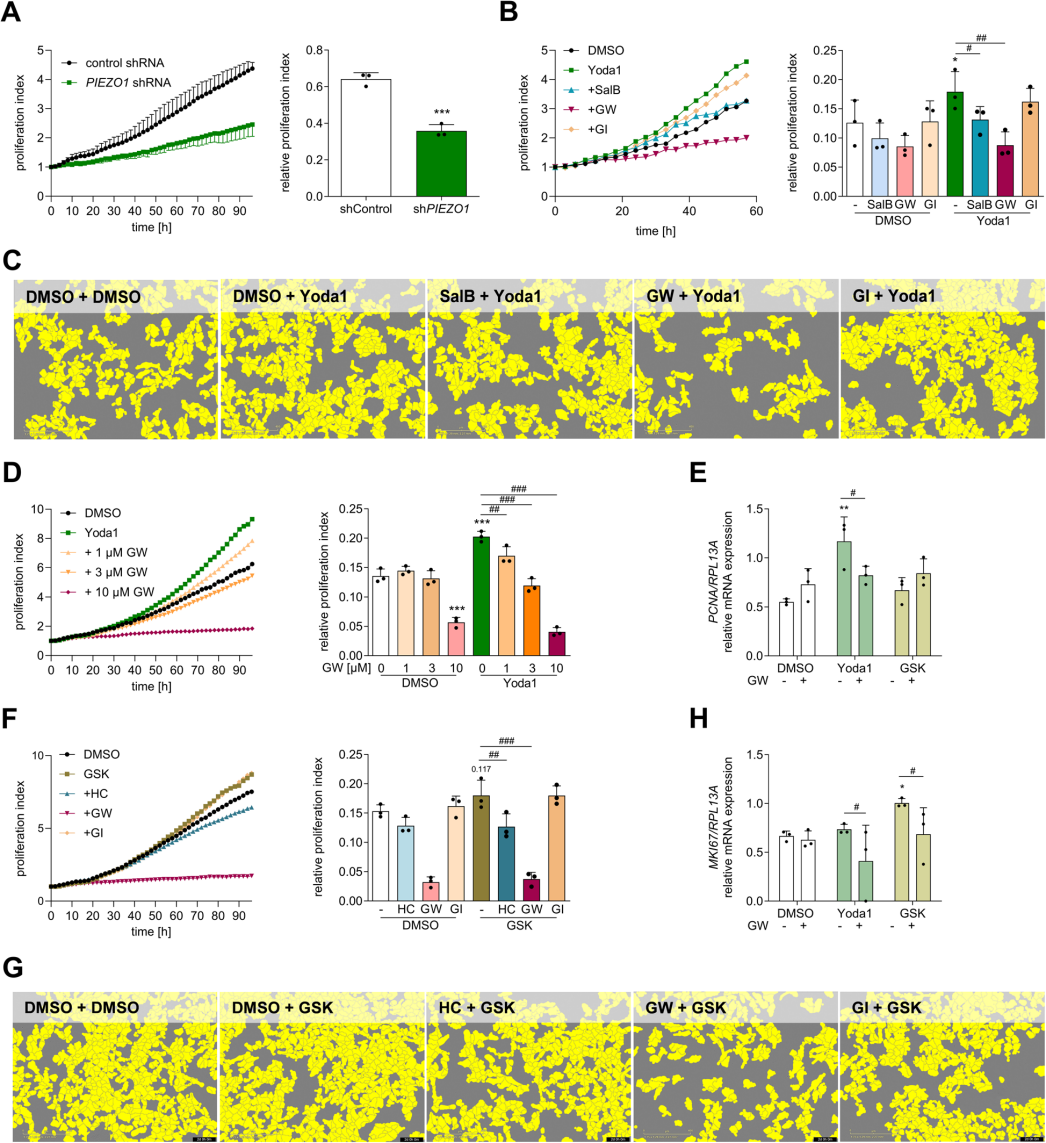
**Piezo1 activation has a pro-proliferative effect on HUVECs mediated by ADAM17.** The proliferation of HUVECs with or without *PIEZO1* knockdown was studied by measuring changes in cell density via live-cell microscopy for up to 96 h and quantified at the 96 h timepoint **A**. Non-transduced HUVECs were treated with or without Yoda1 (0.1 µM) **B**–**E**, **H** or GSK (0.3 nM) **E**–**H**, and cell proliferation was monitored via live-cell microscopy for up to 96 h **B**–**D**, **F**, **G** or via mRNA expression of the proliferation markers *PCNA* **E** and *MKi67* **H** at 24 h time point. The specificity of the ion channels was tested by adding the Piezo1 inhibitor SalB (30 µM) **B**, **C** or the TRPV4 inhibitor HC (0.3 µM) **F**, **G** prior to stimulation. The involvement of ADAMs was tested via the use of the ADAM10 inhibitor GI (10 µM) or the ADAM17/10 inhibitor GW (10 µM) **B**–**H**. The quantification time point for the proliferation experiments with Yoda1 in combination with inhibitors was 48 h **B** and for Yoda1 with GW concentration series **D** and GSK with inhibitors **F** was 96 h. Representative images reflect the 48 h time point **C**, **G**. DMSO was used as a vehicle control. The quantitative data are shown as the means + SDs of at least three independent experiments. Statistical analysis was performed using a generalized linear mixed model (GLMM) with false discovery rate (FDR) correction as post-hoc test. Statistical differences to the control are indicated by asterisks (* *p* ≤ 0.05, ** *p* ≤ 0.01, *** *p* ≤ 0.001), whereas differences between the treatments are indicated by hashes (# *p* ≤ 0.05, ## *p* ≤ 0.01, ### *p* ≤ 0.001)

Endothelial loss of junctional stability affects not only barrier function but also proliferation and migration, which are highly important for vessel repair [39–41]. Therefore, we hypothesized that the axis from Piezo1/TRPV4 to ADAM10 and ADAM17 could be important for the proliferation and migration of endothelial cells. For proliferation assays, HUVECs were seeded at a low density and the change in density was monitored over time up to 96 h via live-cell imaging. First, the effect of Piezo1 was studied. Compared with control cells, *PIEZO1*-knockdown cells showed a significant reduction in proliferation (Fig. 5A), suggesting that Piezo1 is required for cell proliferation even in the absence of an exogenous activator. Next, we stimulated HUVECs with the Piezo1 agonist Yoda1 and observed increased proliferation of HUVECs compared with DMSO-treated controls (Fig. 5B, C). This proliferative effect was blocked by the Piezo1 inhibitor SalB as well as by the ADAM10/ADAM17 inhibitor GW but not by the ADAM10-specific inhibitor GI. Since we also observed a basal inhibitory effect on proliferation by 10 µM GW in the absence of Yoda1, we repeated the experiment with a concentration series of GW and increased the observation time again to 96 h to also see later effects. We found that 3 µM GW was sufficient to completely suppress the proliferative effect in response to Yoda1 without affecting basal proliferation (Fig. 5D). Additionally, expression of proliferating cell nuclear antigen (*PCNA*), a proliferation marker gene, was upregulated after 24 h Yoda1 stimulation, but only to a lesser extend in the presence of GW (Fig. 5E). Next, we studied the influence of TRPV4 activation on endothelial cell proliferation. Interestingly, TRPV4 activation only showed a tendency for increased proliferation at later time points. HC inhibition of TRPV4 had no basal effect but suppressed GSK-stimulated proliferation. As previously described, treatment with GW inhibited proliferation (Fig. 5F, G). Nevertheless, significant changes in mRNA expression of the *MKi67* (marker of proliferation Kiel 67) could be found after 24 h GSK stimulation (Fig. 5H). These data suggest that Piezo1 stimulation has a more pronounced effect on proliferation compared to TRPV4 stimulation. Moreover, as suggested by the inhibition profile of GI and GW, Piezo1 seems to induce proliferation in an ADAM17- but not ADAM10-dependent manner.

**Fig. 6:**
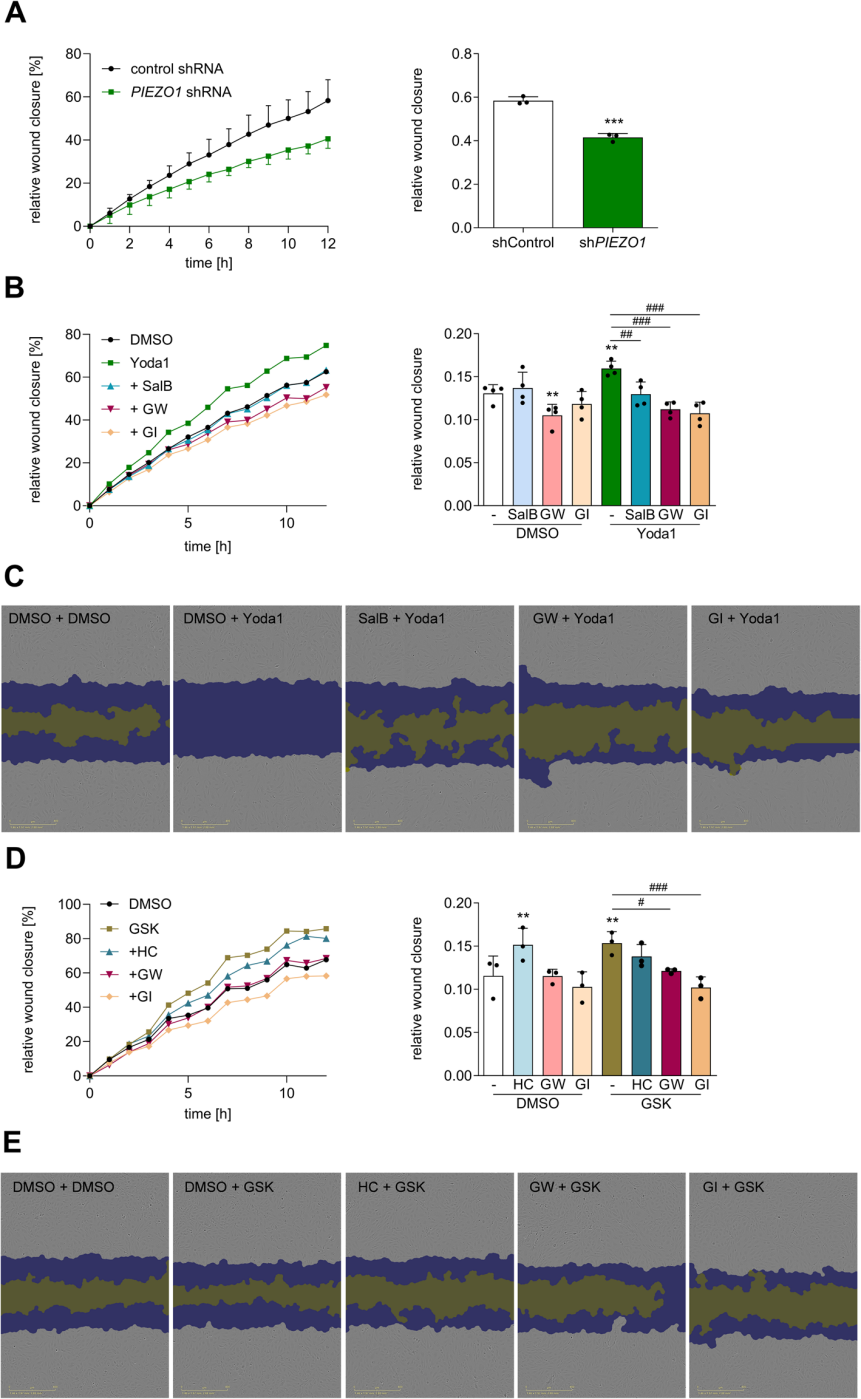
**Piezo1 activation has a pro-migratory effect on HUVECs mediated by ADAM10**. The migration of HUVECs with or without shRNA-mediated PIEZO1 knockdown **A** was studied by scratching identical wounds into monolayers and monitoring wound closure over 12 h via live-cell microscopy. HUVECs were studied for wound closure in response to either 0.03 µM Yoda1 to activate Piezo1 **B**, **C** or 0.3 nM GSK to activate TRPV4 **D**, **E**. The specificity of the ion channels was tested by adding the Piezo1 inhibitor SalB (30 µM) **B**, **C** or the TRPV4 inhibitor HC (0.3 µM) **D**, **E** prior to stimulation. The involvement of ADAMs was tested using the ADAM10 inhibitor GI (10 µM) or the ADAM17/10 inhibitor GW (10 µM) **B**–**D**. **C** and **E** display representative images for wound closure, showing the initial wound (purple) and the current wound (yellow) at the time point showing the greatest effect (11 h for Yoda1 and 5 h for GSK). DMSO was used as a vehicle control. Quantification of the data was performed at the 10 h time point. The quantitative data are shown as the means + SDs of at least three independent experiments. Statistical analysis was performed using a generalized linear mixed model (GLMM) with false discovery rate (FDR) correction as post-hoc test. Statistical differences to the control are indicated by asterisks (* *p*≤0.05, ** *p*≤0.01, *** *p*≤0.001), whereas differences between the treatments are indicated by hashes (# *p*≤0.05, ## *p*≤0.01, ### *p*≤0.001)

To investigate the migratory behavior of endothelial cells, a wound was scratched into a cell monolayer of HUVECs, and migration-based wound closure was monitored over time. As no significant effects on proliferation were observed within 12 h (Fig. 5B, D or Suppl. Figure 12), we performed the migration assay without the addition of a proliferation inhibitor such as mitomycin, in order to minimize additional cellular stress. Compared with control cells, *PIEZO1*-knockdown cells presented a profoundly diminished wound closure response (Fig. 6A). Vice versa, stimulation of HUVECs with Yoda1 increased wound closure, and this effect was suppressed by treatment with the Piezo1 inhibitor SalB (Fig. 6B, C). To study the role of ADAM proteases in this wound closure response, the ADAM inhibitors GW and GI were applied. Interestingly, the ADAM10-specific inhibitor GI was sufficient to block Yoda1-induced cell migration completely without affecting the basal migration of unstimulated cells (Fig. 6B, C). Finally, the influence of TRPV4 on wound closure was investigated. The GSK-mediated activation of TRPV4 seemed to enhance cell migration, but the TRPV4 inhibitor HC alone had a similar effect on unstimulated cells, but not in combination with GSK. In both cases, ADAM10 inhibition by GI was sufficient to reduce the migratory response (Fig. 6B-E).

Taken together, these findings indicate that Piezo1 activation promotes endothelial migration, and this response seems to depend on ADAM10. For TRPV4, the data are less consistent since both TRPV4 activation and inhibition induce a migratory response.

## Discussion

In this study, we provide multiple lines of evidence that the mechanosensitive ion channel Piezo1 and the polymodal ion channel TRPV4 can induce the activation of the two most prominent ADAM family members, ADAM17 and ADAM10, in endothelial cells. Mechanical or pharmacological stimulation of either channel leads to the release of the ADAM substrates JAM-A and TNFR1 as well as the ADAM-dependent loss of VE-cadherin at endothelial junctions. While TRPV4-mediated ADAM activation seems to have only moderate impact on the regulation of endothelial functions, Piezo1-induced activation of ADAM10 or ADAM17 is crucial for endothelial proliferation and migration.

Many mechanosensors, including ion channels (Piezo1, TRP channels, Kir2.1, epithelial sodium channel ENaC, and potassium channel subfamily K member 2 TREK-1), receptors (G protein-coupled receptors (GPCR), receptor tyrosine kinases), and membrane structures (caveolae, PECAM1, PlexinD1, glycocalyx, cadherins, integrins, and cilia), are known to sense mechanical forces in endothelial cells. Here, we show that both Piezo1 and TRPV4 are involved in shear-induced activation of ADAM17 and ADAM10 in endothelial cells. Nevertheless, there is evidence that ADAM activity in response to mechanical forces is not limited to mechanotransduction mediated by the ion channels Piezo1 and TRPV4 [25, 26, 42–44] but also involves interactions with integrins [45, 46] or GPCRs [47]. The variety of distinct activation mechanisms could explain the partial effects on shear stress-mediated ADAM activation via *PIEZO1* knockdown or the inhibition of Piezo1 with SalB, TRPV4 with HC or ion channels with GsMTx4. However, with the use of the well-established Piezo1 agonist Yoda1 and the TRPV4 agonist GSK, which showed clear effects for the channel inhibitors and knockdown cells, we provide strong evidence that the association of Piezo1 and TRPV4 activation with the induction of ADAM activity under mechanical conditions is an important control mechanism for the regulation of endothelial homeostasis.

It has been proposed that Piezo1 can act as an upstream activator of TRPV4 in HUVECs [14]. However, in another study, both channels act independently in aortic endothelial cells [48] whereas a further study not only found that the two channels act independently but even induced opposing effects in the portal vein [15]. Here, we demonstrate that, for HUVECs, both channels are capable of activating ADAMs to a comparable extent but independently. This discrepancy may be attributed to the different readouts employed in the three studies, as well as variations in the primary cells from different origins.

For endothelial cells Piezo1-induced transcriptional regulation of shear stress responding genes such as *KLF2* and target genes of the Notch1 pathway has been reported several times before [49–51]. Our study revealed that both mechanical activation by shear stress and pharmacological activation with Yoda1 lead to changes in not only the transcriptional level but also the posttranslational level through the release of increased amounts of the ADAM substrates JAM-A, VE-cadherin and TNFR1 by HUVECs. This Piezo1-mediated mechanism includes the activation of both ADAM10 and ADAM17. Another report showed ADAM10-dependent activation of the Notch1 signaling pathway induced by shear stress and Yoda1 in HMVECs (human cardiac microvascular endothelial cells) [25]. This finding was also confirmed in our study with HUVECs and could also be extended to ADAM17 activation via the cleavage of the ADAM17 substrates JAM-A and TNFR1. Notably, ADAM17 activation was also linked to Piezo1, as an important part of the angiopoietin/TIE/FOXO1 signaling pathway, in lymphatic endothelial cells [44]. Moreover, we recently reported increased growth factor shedding and remodeling of tight and adherens junctions as a result of Piezo1-dependent ADAM10 and ADAM17 activation in epithelial H441 cells induced by Yoda1 and stretch [26]. Additionally, besides Piezo1-mediated effects on ADAM activation, mechanical and pharmacological induction of TRPV4 activity leads not only to ADAM10 but also to ADAM17 activation, resulting in the release of soluble JAM-A and TNFR1. To date, only ADAM10-mediated shedding of the adhesion proteins epithelial cadherin (E-cadherin) and CD44 induced by TRPV4 has been shown in pulmonary alveolar type 1 (AT1) cells and chondrocytes [42, 43]. This effect is physiologically correlated with the regulation of endothelial and epithelial barrier integrity. In addition, ADAM-induced shedding of surface molecules impacts several endothelial functions, especially those involved in inflammation and repair. On the one hand, soluble JAM-A is important for regulating leukocyte transmigration by interacting with receptors on immune cells [52] or influencing barrier function by binding to adhesion partners. On the other hand, soluble TNFR1 can act as an anti-inflammatory agent through the capture of circulating TNFα before binding to the cell surface, preventing the subsequent inflammatory response or inducing apoptosis [53, 54]. This finding is consistent with the observation that the activation of ADAM10 or ADAM17 can have very different effects in an inflammatory setting, depending on the presence and shedding of specific substrates for proteases [55].

The effects of Piezo1 and TRPV4 on adhesion molecules such as VE-cadherin and PECAM1 without any linkage to ADAM proteases have been shown several times under flow conditions as well as with Yoda1 or GSK [14, 16, 56, 57]. Here, we explain and extend these findings by showing that the Piezo1-induced loss of VE-cadherin at junctions is at least partially mediated through the activation of ADAM proteases. Furthermore, Yoda1 and GSK also induce an initial ADAM-dependent reduction of surface expressed VE-cadherin as well as of the total cellular content. This was not compensated by induced VE-cadherin mRNA expression. However, surface levels of VE-cadherin could be restored at later time points. Since there was no regulation on the transcriptional level this might be due to constitutive VE-cadherin production. In addition to its function as an adapter in the mechanosensory complex [58], it is, like JAM-A, strongly involved in the regulation of vascular permeability and leukocyte extravasation due to its adhesion capacity. Moreover, PECAM1 was shown to support leukocyte diapedesis via tension-dependent phosphorylation of VE-cadherin [59]. Weakening of the junctions, as we showed in this study, is associated with inflammatory processes, including proliferation or migration, linking VE-cadherin and JAM-A not only to an adhesion task but also to cellular responses [60–63]. Since we could show an ADAM-dependent positive regulation of migration and proliferation following Piezo1 activation, this could be a possibility how the Piezo1-ADAM axis contributes to these functional processes.

Additionally, the transcriptional regulation induced by the Yes-associated protein/transcriptional coactivator with PDZ-binding motif (YAP/TAZ), growth factor signaling and ADAM-mediated shedding of Notch or JAM-A, which are known to be involved in regulating functional processes, represents additional possible interfaces between Piezo1/TRPV4 and ADAM signaling [45, 52, 62, 64–68]. This complexity in the regulation of cellular functions suggests that the observed effects might not be mediated by only one pathway in all cell types; instead, different pathways may be implicated in different cell types. For example, TRPV4 is reported to play an important role in breast cancer cell migration via Ca^2+^-dependent activation of protein kinase B (AKT) and downregulation of E-cadherin [69]. However, in this study, we were unable to confirm a similar role for TRPV4 in regulating endothelial proliferation and migration, which may suggest distinct regulatory functions of the two channels. This might also be reflected by the differential expression pattern of *PCNA* and *MKI67* which could indicate that the cells are in different cell cycle phases [70]. Nonetheless, it should be noted that, particularly with regard to proliferation, the effectiveness of the inhibitors used may have declined over time, potentially resulting in a false-negative outcome for TRPV4. In addition, we used a substantially lower concentration than reported in the literature, as we observed clear signs of cytotoxicity at higher concentrations. This discrepancy may be explained by the fact that many previous studies with endothelial cells focused primarily on short-term calcium measurements, during which effects on cell viability are not yet apparent. Furthermore, donor-specific variability could also explain these differences in GSK sensitivity. In our own experiments, we already observed slight differences in the tolerance to Yoda1 and GSK between the various donors included in this study.

The involvement of calcium-dependent PKC in ADAM17 activation was reported early on, particularly through the use of the PKC activator PMA [27]. In the present study, we were able to exclude a role for PKC in Piezo1/TRPV4-mediated ADAM activation by using the pan-PKC inhibitor BIM-2. This finding strongly suggests that an entirely different regulatory mechanism is at play, which warrants further investigation in future studies. A plausible alternative could involve other kinases, such as the calcium-dependent kinase CaMKII, as well as AKT, ERK1/2, or FAK, as all of them have been shown to be activated downstream of Piezo1 activation [67, 71–73]. ERK1/2 appears particularly promising in this context, as it has been shown to interact with the ADAM17 regulator iRhom2, suggesting a potential role in ADAM17 activation [74]. The relevance of those kinase-related pathways could not be confidently confirmed in this study, yet. The underlying mechanism displays high complexity indicating the involvement of several pathways connecting Piezo1 activation to increased ADAM activity. Another regulatory mechanism that may be involved is a phosphatidylserine (PS) switch. On the one hand, Piezo1 can be desensitized via a PS switch [75]. On the other hand, ADAM10 and ADAM17 can be activated by reversible PS exposure [76, 77]. It is therefore possible that Piezo1 activation induces a transient PS switch that limits further Piezo1 activation while promoting ADAM activity. Once membrane asymmetry is restored, the pathway could be reactivated. This would represent a self-regulatory mechanism to prevent excessive ADAM activation in response to mechanical stimuli. Moreover, this type of non-apoptotic PS externalization has been described in lymphocytes following activation of the purinergic receptor P2X7 [78]. This presents an additional layer of potential regulation, as Piezo1-mediated ATP release through pannexin and subsequent paracrine activation of purinergic receptors has already been reported in lung epithelial cells [79]. These receptors could, in turn, contribute to the activation of ADAM proteases, as purinergic receptor involvement in ADAM activation has also been described [80].

Although shear stress plays a central role in regulating endothelial homeostasis and was therefore the main focus of this study, other mechanical forces also contribute to endothelial function. One important factor to consider is the composition and, consequently, the stiffness of the extracellular matrix (ECM). Interestingly, both ECM composition and stiffness can directly influence the sensitivity of Piezo1 [81]. Alterations in substrate stiffness or ECM composition could therefore modify the effects observed in this study, as has already been demonstrated for the impact of stretch on endothelial cells [82]. In our experimental setup, endothelial cells were cultured on rigid tissue culture plastic, which may represent a pro-atherosclerotic phenotype as it induces a similar signaling pattern as fibronectin [81, 83, 84]. It is also important to note that matrix metalloproteinases (MMPs) can actively modulate ECM properties [85]. While MMPs are primarily responsible for this remodeling, ADAM10 and ADAM17 can also influence the glycocalyx by shedding syndecans [86]. This modification of the glycocalyx may affect cellular mechanosensing, as the glycocalyx itself has been proposed as a mechanosensory structure [87, 88]. Moreover, it may directly interact with ion channels [89].

Having now demonstrated the influence of mechanosensitive ion channels on ADAM10 and ADAM17 activity in both endothelial and epithelial cells [26], and given that supporting findings in similar or different cell types have been reported [25, 42–44], the Piezo1/TRPV4–ADAM axis may represent a generalizable mechanism by which mechanical forces are rapidly translated into cellular responses across various cell types. It would be particularly interesting to investigate whether this axis also plays a role in immune cells such as monocytes and neutrophils, which are key contributors to the development of inflammatory diseases such as atherosclerosis and acute respiratory distress syndrome (ARDS). Neutrophils appear to be especially relevant in this context, as all components of the axis have been implicated in the regulation of neutrophil function [90–94].

In conclusion, our data provide clear evidence that a direct link between ion channel activation of Piezo1 and TRPV4 mediates the activation of the metalloproteases ADAM17 and ADAM10, affecting vascular endothelial cell functions in response to mechanical stimuli. This process makes ADAM proteases an important class of secondary mechanoresponders. This response to mechanical stimuli influences cell integrity through the loss of cell‒cell junctions as well as the migratory and proliferative behavior of endothelial cells. Furthermore, these findings implicate the involvement of the Piezo1/TRPV4-ADAM axis in the regulation of inflammatory processes, by controlling endothelial permeability after mechanical changes. Therefore, it is important to further investigate the signaling cascade within the axis to better understand the interaction and to identify potential therapeutic targets. Beyond the investigated substrates, studying other key ADAM substrates involved in inflammatory processes could help to unravel the role of the studied mechanotransduction axis in vascular integrity, trans-endothelial migration and inflammatory crosstalk.

## Supplementary Information

Supplementary Material 1.

## Data Availability

No datasets were generated or analysed during the current study.
