# Supplementary material for: Mechanoresponsive ion channels Piezo1 and TRPV4 stimulate ADAM10 and ADAM17 with differential impact on endothelial migration and proliferation
Source: Cell Commun Signal. 2026 Jan 10;24:52. doi: 10.1186/s12964-025-02633-x (PMC12849641; doi:10.1186/s12964-025-02633-x)
Supplement: Supplementary file 1 — Supplementary Material 1. [file 12964_2025_2633_MOESM1_ESM.pdf]

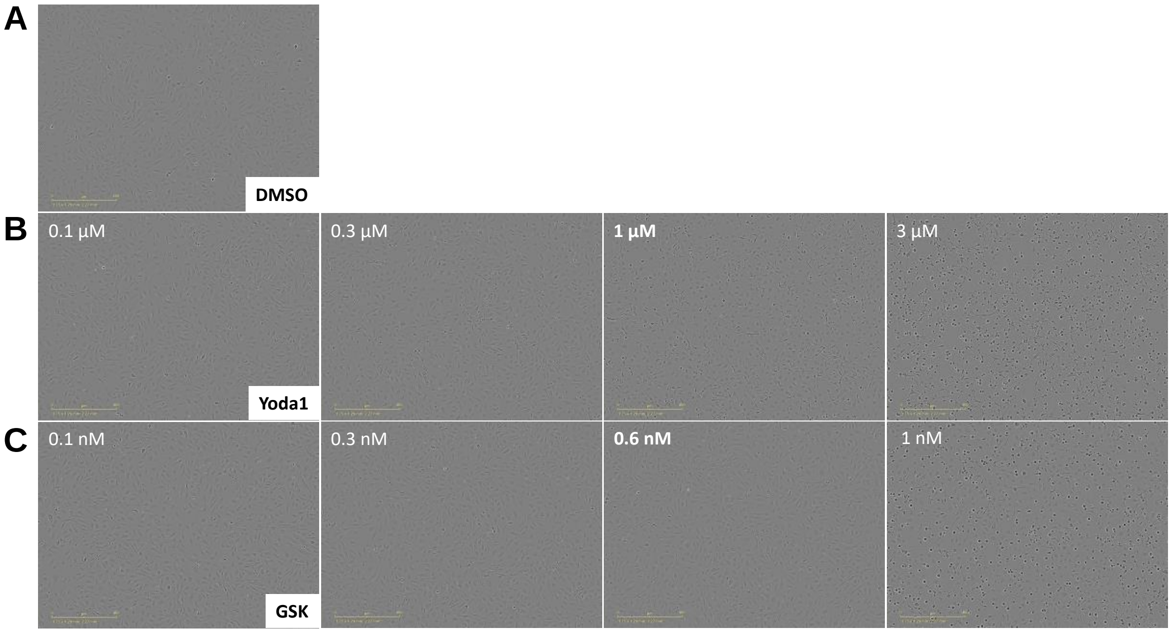

Suppl. Fig. 1: Concentration series of Piezo1 agonist Yoda1 and TRRPV4 agonist GSK.

HUVECs were treated with DMSO according to the highest volume of agonist used **A**, 0.1 – 3 µM Yoda1 **B**, or 0.1 - 1 nM GSK for 0.5 h **C**. Images were taken with the Incucyte SX5.

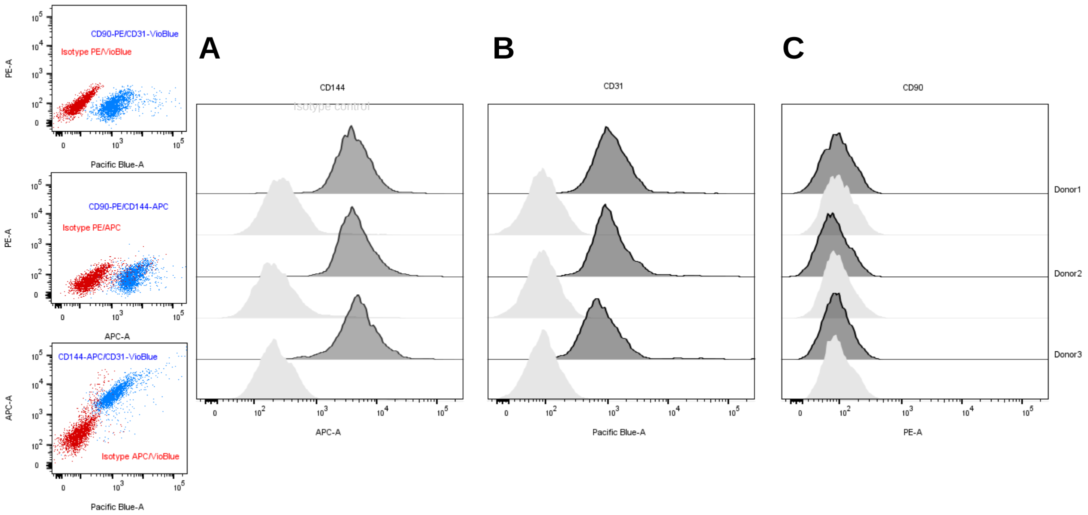

Suppl. Fig. 2: Proof of purity of the used HUVEC cultures.

HUVECs from three different donors were tested for typical endothelial surface proteins CD144 (VE-cadherin) **A**, CD31 (PECAM-1) **B**, and the fibroblast marker CD90 (Thy-1) **C** compared to the according isotype control using flow cytometry.

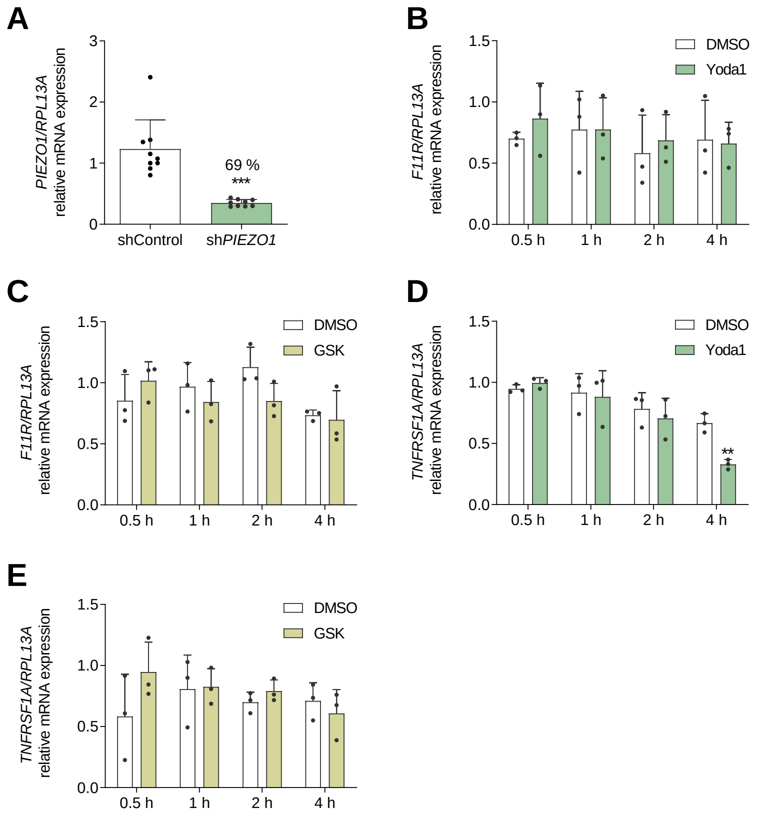

Suppl. Fig. 3: Piezo1 activation mediates no change on the transcriptional level of *JAM-A* and *TNFR1.*

HUVECs were treated with 1 µM Yoda1 or 0.6 nM GSK for 0.5, 1, 2, or 4 h. *F11R (JAM-A)* **A, B** and *TNFRSF1A (TNFR1)* **C, D** mRNA expression was measured in relation to the expression of the reference gene *RPL13A* in the cell lysates and presented as relative mRNA expression. Quantitative data are shown as mean + SD of at least three independent experiments. Statistical analysis was performed using a generalized linear mixed model (GLMM) with false discovery rate (FDR) correction as post-hoc test. Statistical differences to the control are indicated by asterisks (* p≤0.05, ** p≤0.01, *** p≤0.001).

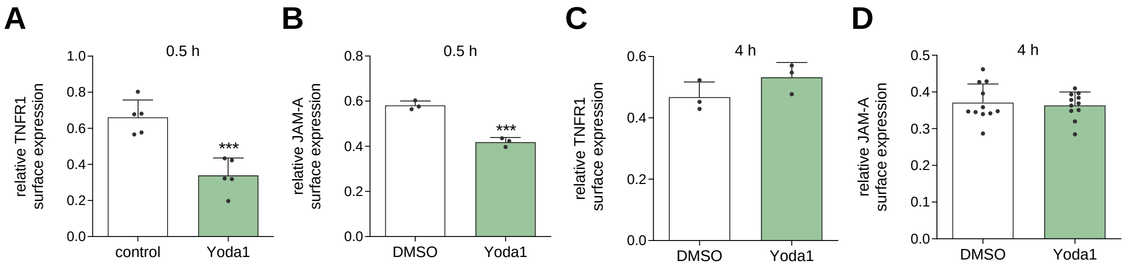

Suppl. Fig. 4: Influence of Piezo1 activation on TNFR1 and JAM-A surface levels.

HUVECs were treated with Yoda1 (1 µM) for 0.5 h and 4 h. The surface expression of TNFR1 **A**, **C** and JAM-A **B**, **D** was analyzed via flow cytometry. Quantitative data are shown as mean + SD of at least three independent experiments. Statistical analysis was performed using a generalized linear mixed model (GLMM) with false discovery rate (FDR) correction as post-hoc test. Statistical differences to the control are indicated by asterisks (* p≤0.05, ** p≤0.01, *** p≤0.001).

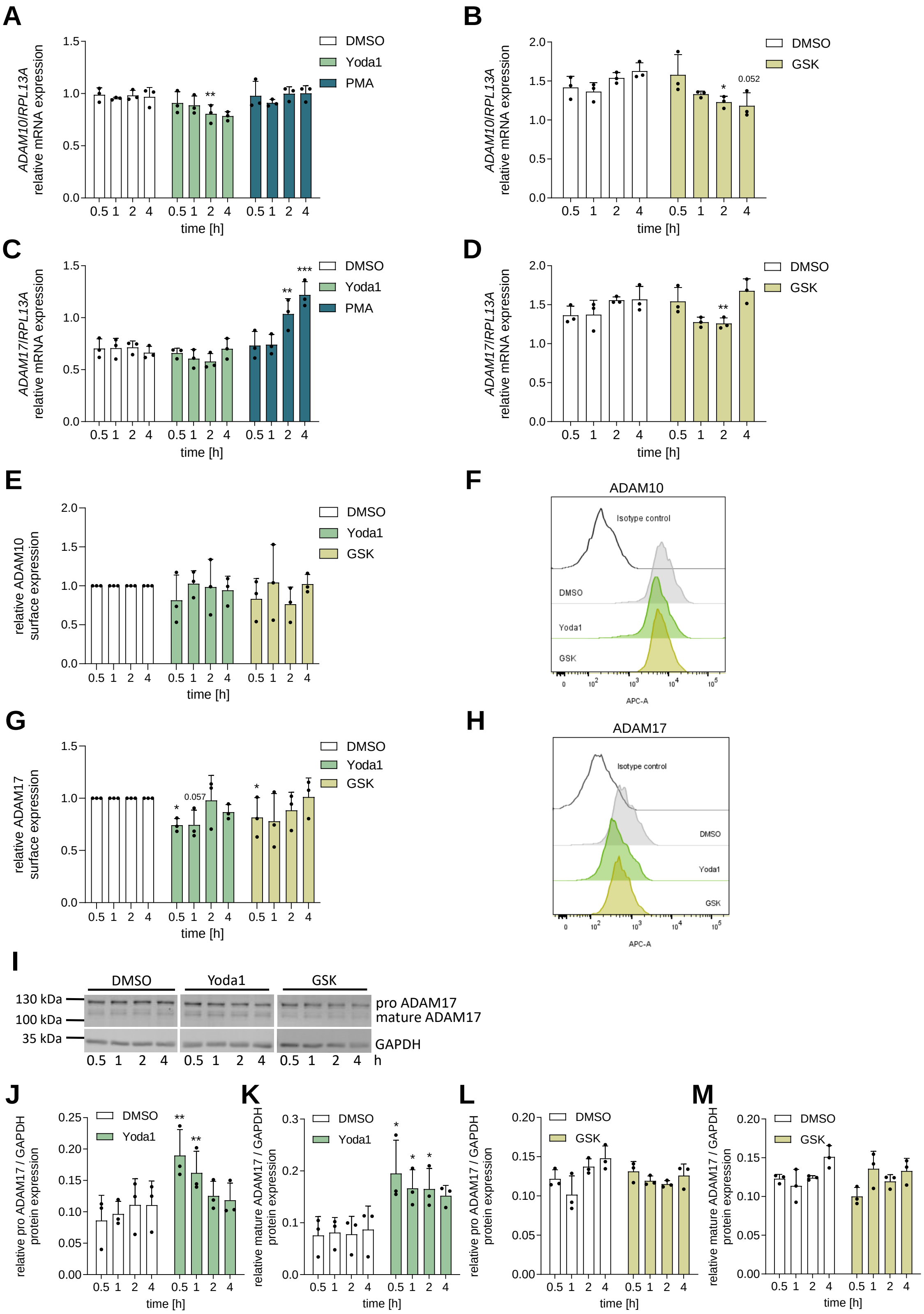

Suppl. Fig. 5: Influence of Piezo1 and TRPV4 activation on ADAM10/17 expression.

HUVECs were treated with Yoda1 (1 µM), PMA (0.1 µM) or GSK (0.6 nM) for 0.5 h, 1 h, 2 h or 4 h. *ADAM10* **A**, **B** and *ADAM17* **C**, **D** mRNA expression was measured in relation to the expression of the reference gene *RPL13A* in the cell lysates and presented as relative mRNA expression. The surface expression of ADAM10 **E**, **F** and ADAM17 **G**, **H** was investigated by flow cytometry using according

isotypes as control. Representative flow cytometric curves showing measurement of timepoint 0.5h **F**,**H**. Pro ADAM17 **I**, **J**, **L** and mature ADAM17 **I**, **K**, **M** protein expression levels were determined via western blotting of the whole cell lysate. GAPDH served as loading control. Quantitative data are shown as mean + SD of at least three independent experiments. Statistical analysis was performed using a generalized linear mixed model (GLMM) with false discovery rate (FDR) correction as post-hoc test. Statistical differences to the control are indicated by asterisks (* p≤0.05, ** p≤0.01, *** p≤0.001).

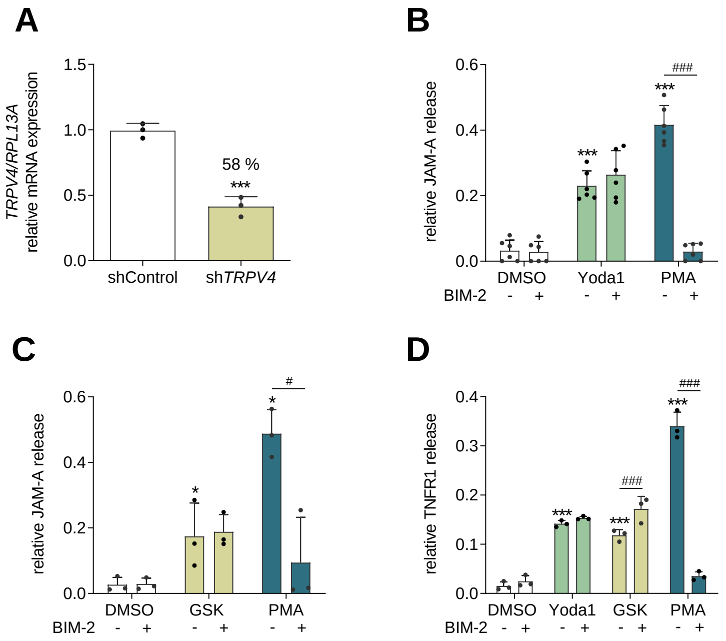

Suppl. Fig. 6: Piezo1- and TRPV4-mediated ADAM activation is independent of PKC signaling.

The knockdown efficiency of *TRPV4* mRNA expression in shControl and sh*TRPV4* cells was normalized to the corresponding mRNA expression of the reference gene *RPL13A* and presented as relative mRNA expression **A**. HUVECs were pre-incubated with BIM-2 (1 µM), followed by addition of Yoda1 (1 µM) **A, C** or GSK (0.6 nM) **B**, **C** and further incubation for 4 h. Concentrations of soluble JAM-A **B** and **C**, or TNFR1 **D**, were determined via ELISA. Quantitative data are shown as mean + SD of at least three independent experiments. Statistical analysis was performed using a generalized linear mixed model (GLMM) with false discovery rate (FDR) correction as post-hoc test. Statistical differences to the control are indicated by asterisks (* p≤0.05, ** p≤0.01, *** p≤0.001), while differences between the treatments are indicated by hashes (# p≤0.05, ## p≤0.01, ### p≤0.001).

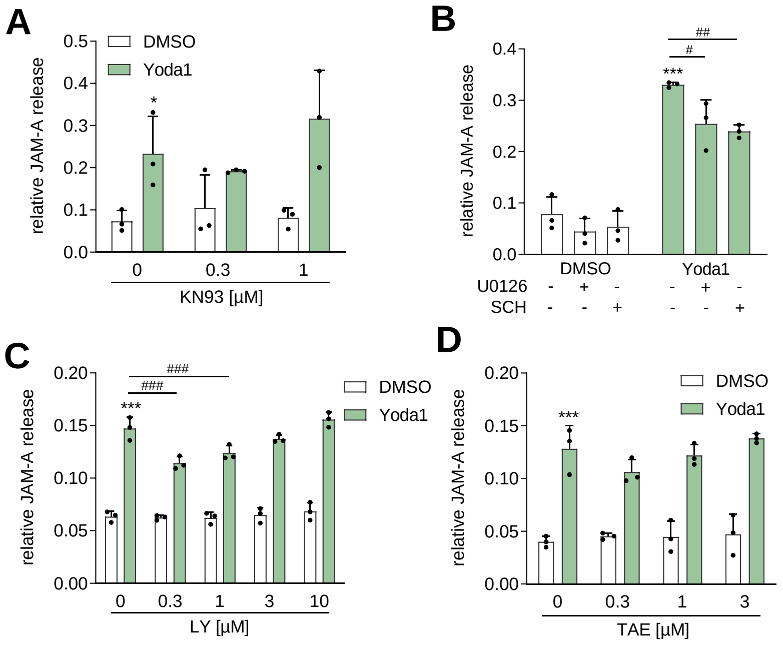

Suppl. Fig. 7: Involvement of kinases in the Piezo1-mediated JAM-A release.

HUVECs were pre-incubated with 0.3 µM and 1 µM CaMKII inhibitor KN93 **A**, 0.3 µM MEK1/2 inhibitor U0126 and 1 µM ERK1/2 inhibitor SCH772984 (SCH) **B**, 0.3 – 10 µM PI3K inhibitor LY294002 (LY) **C** and 0.3 – 3 µM FAK inhibitor TAE226 (TAE) **D** for 1 h, followed by addition of Yoda1 (1 µM) and further incubation for 4 h. Concentrations of soluble JAM-A were determined via ELISA. Quantitative data are shown as mean + SD of at least three independent experiments. Statistical analysis was performed using a generalized linear mixed model (GLMM) with false discovery rate (FDR) correction as post-hoc test. Statistical differences to the control are indicated by asterisks (* p≤0.05, ** p≤0.01, *** p≤0.001), while differences between the treatments are indicated by hashes (# p≤0.05, ## p≤0.01, ### p≤0.001).

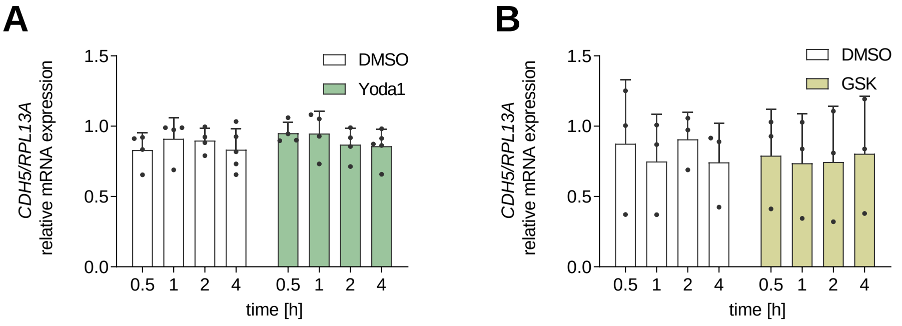

Suppl. Fig. 8: Influence of Piezo1 and TRPV4 activation on *CDH5* expression levels.

HUVECs were treated with Yoda1 (1 µM) **A** or GSK (0.6 nM) **B** for 0.5 h, 1 h, 2 h, or 4 h. *CDH5* (VE-cadherin) mRNA expression was measured in relation to the expression of the reference gene *RPL13A* in the cell lysates and presented as relative mRNA expression. Quantitative data are shown as mean + SD of at least three independent experiments. Statistical analysis was performed using a generalized linear mixed model (GLMM) with false discovery rate (FDR) correction as post-hoc test. Statistical differences to the control are indicated by asterisks (* p≤0.05, ** p≤0.01, *** p≤0.001).

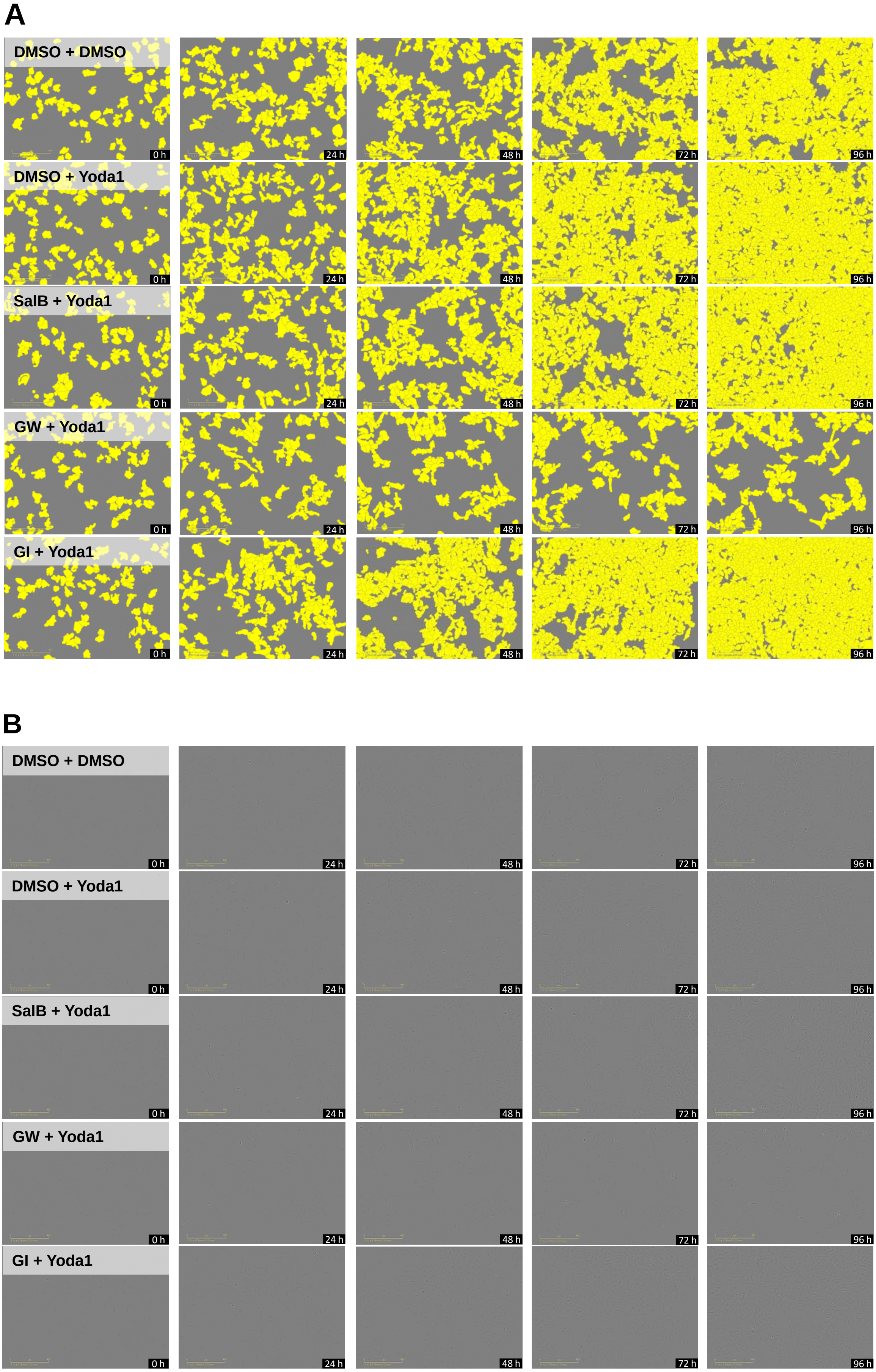
A
DMSO + DMSO
DMSO + Yoda1
SalB + Yoda1
GW + Yoda1
GI + Yoda1
0 h
24 h
48 h
72 h
96 h
B
DMSO + DMSO
DMSO + Yoda1
SalB + Yoda1
GW + Yoda1
GI + Yoda1
0 h
24 h
48 h
72 h
96 h

Suppl. Fig. 9: Piezo1 activation has a pro-proliferative effect on HUVECs mediated by ADAM17.

HUVECs were pre-incubated with the Piezo1 inhibitor SalB (30 µM), the ADAM10/17 inhibitor GW (10 µM) and the ADAM10 inhibitor GI (10 µM) for 1 h, followed by addition of Yoda1 (1 µM). DMSO was used as a vehicle control. The proliferation was monitored by measuring changes in cell density via live-cell microscopy for up to 96 h using the analysis tool of the Incucyte SX5. The representative images showing the different conditions in 24 h intervals with **A** or without **B** the used analysis mask for cell proliferation quantification (shown in yellow).

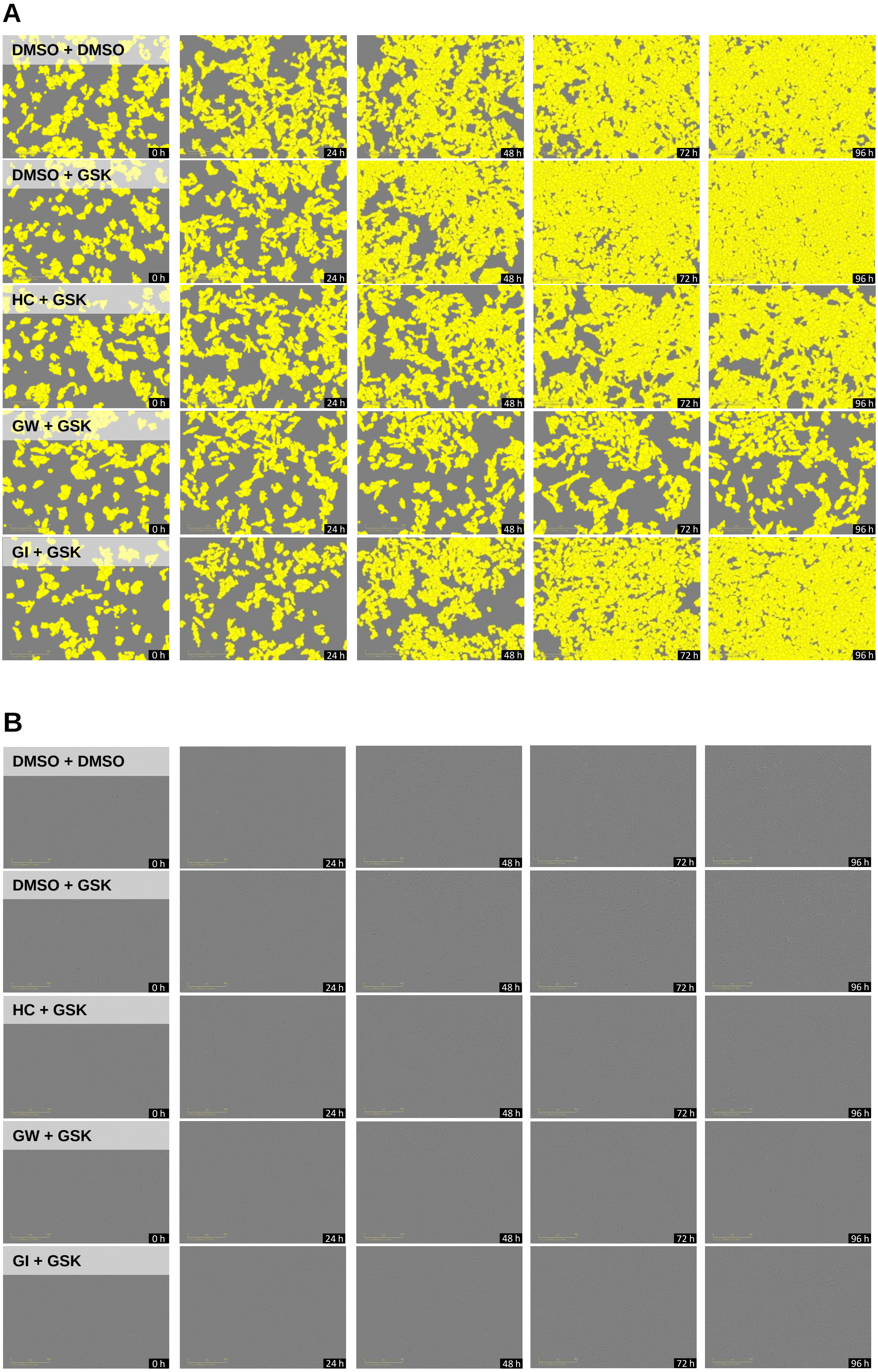
A
DMSO + DMSO
DMSO + GSK
HC + GSK
GW + GSK
GI + GSK
0 h
24 h
48 h
72 h
96 h
B
DMSO + DMSO
DMSO + GSK
HC + GSK
GW + GSK
GI + GSK
0 h
24 h
48 h
72 h
96 h

Suppl. Fig. 10: TRPV4 activation has a slight pro-proliferative effect on HUVECs mediated by ADAM17.

HUVECs were pre-incubated with the TRPV4 inhibitor HC (0.3 µM), the ADAM10/17 inhibitor GW (10 µM) and the ADAM10 inhibitor GI (10 µM) for 1 h, followed by addition of GSK (0.6 nM). DMSO was used as a vehicle control. The proliferation was monitored by measuring changes in cell density via live-cell microscopy for up to 96 h using the analysis tool of the Incucyte SX5. The representative images showing the different conditions in 24 h intervals with **A** or without **B** the used analysis mask for cell proliferation quantification (shown in yellow).

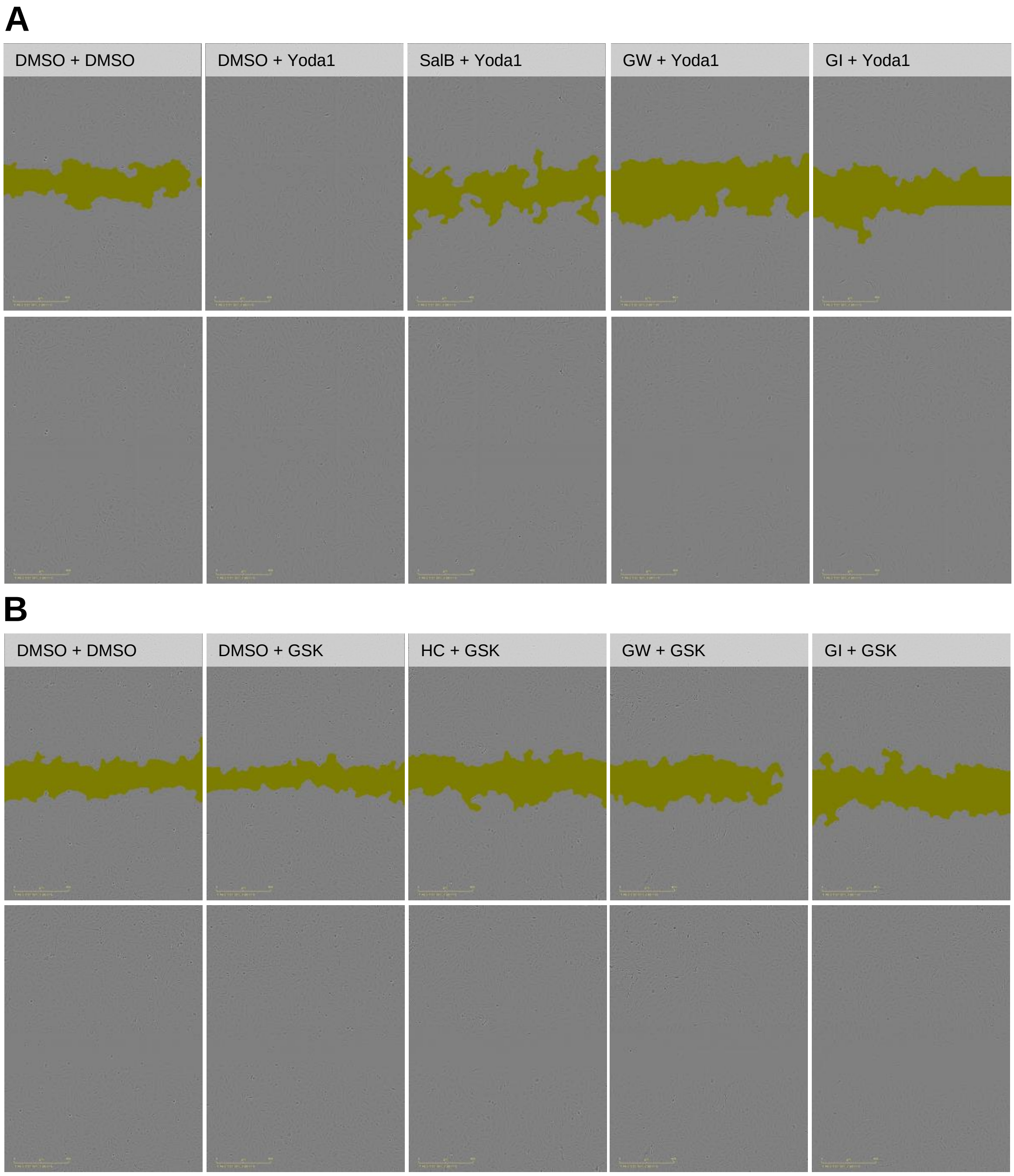

Suppl. Fig. 11: Piezo1 and TRPV4 activation have pro-migratory effects on HUVECs mediated by ADAM10.

HUVECs were pre-incubated with the Piezo1 inhibitor SalB (30 µM) **A** or TRPV4 inhibitor HC (0.3 µM) **B**, the ADAM10/17 inhibitor GW (10 µM) and the ADAM10 inhibitor GI (10 µM) for 1 h, followed by addition of Yoda1 (1 µM) **A** or GSK (0.6 nM) **A**. DMSO was used as a vehicle control. The migration was monitored by measuring changes in wound closure via live-cell microscopy for up to 12 h using the analysis tool of the Incucyte SX5. The representative images showing the different stimulations with or without the analysis mask for wound closure quantification at time points of greatest effect (shown in yellow).

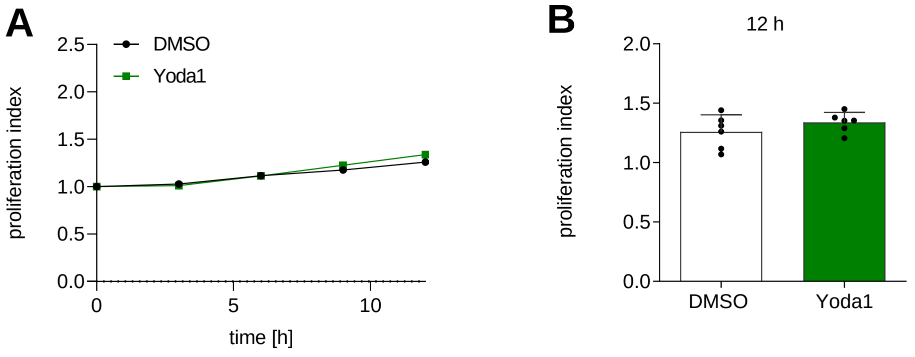

Suppl. Fig. 12: Piezo1 activation has no pro-proliferative effects on HUVECs within 12 hours.

HUVECs were treated with or without Yoda1 (1 µM) and changes in cell density were measured using live-cell microscopy for up to 12 h. DMSO was used as vehicle control. The proliferation index was calculated as the ratio of the counted number of cells at a given time point to the number of cells at the 0 h time point. The quantitative data are shown as the means + SDs of at least three independent experiments. Statistical analysis was performed using a generalized linear mixed model (GLMM) with false discovery rate (FDR) correction as post-hoc test. Statistical differences to the control are indicated by asterisks (* p≤0.05, ** p≤0.01, *** p≤0.001).

**Figure 1: Flow-induced ADAM activation involves Piezo1 and TRPV4**					
**A**			**B**		
0 dyne/cm2	DMSO	39.65 ± 8.67	0 dyne/cm2	DMSO	5.84 ± 3.67
0 dyne/cm2	GW	33.44 ± 10.58	0 dyne/cm2	GW	3.52 ± 4.4
15 dyne/cm2	DMSO	177.30 ± 30.83	15 dyne/cm2	DMSO	47.52 ± 6.73
15 dyne/cm2	GW	49.06 ± 14.56	15 dyne/cm2	GW	12.54 ± 7.42
**C**			**E**		
0 dyne/cm2	DMSO	129.91 ± 73.07	0 dyne/cm2	shControl	109.94 ± 59.39
0 dyne/cm2	SalB	150.04 ± 57.17	0 dyne/cm2	sh*PIEZO1*	212.62 ± 89.19
15 dyne/cm2	DMSO	289.6 ± 26.02	15 dyne/cm2	shControl	501.51 ± 392.54
15 dyne/cm2	SalB	207.99 ± 61.86	15 dyne/cm2	sh*PIEZO1*	629.68 ± 497.62
**F**			**G**		
0 dyne/cm2	DMSO	110.75 ± 65.86	0 dyne/cm2	DMSO	131.05 ± 47.37
0 dyne/cm2	HC	108.73 ± 60.63	0 dyne/cm2	GsMTx4	126.63 ± 57.67
15 dyne/cm2	DMSO	322.87 ± 90.37	15 dyne/cm2	DMSO	746.99 ± 121.43
15 dyne/cm2	HC	224.29 ± 52.99	15 dyne/cm2	GsMTx4	449.79 ± 127.21

Supp. table 1: ELISA mean raw values of Figure 1 in pg/ml.

**Figure 2: Release of ADAM10- & ADAM17-specific substrates is induced by chemical activation of Piezo1 and TRPV4**

C			D		
0.5 h	DMSO	13.17 ± 9.75	0.5 h	DMSO	50.59 ± 1
0.5 h	Yoda1	12.12 ± 4.3	0.5 h	GSK	66.78 ± 6.48
0.5 h	PMA	31.16 ± 11.26	0.5 h	PMA	81.48 ± 3.97
1 h	DMSO	12.63 ± 7.78	1 h	DMSO	55.78 ± 3.17
1 h	Yoda1	25.36 ± 12.97	1 h	GSK	132.92 ± 22.07
1 h	PMA	88.22 ± 40.45	1 h	PMA	190.12 ± 24.13
2 h	DMSO	8.9 ± 6.49	2 h	DMSO	60,42 ± 3.63
2 h	Yoda1	49.13 ± 14.03	2 h	GSK	717.28 ± 38.27
2 h	PMA	174.58 ± 80.39	2 h	PMA	300.3 ± 70.77
4 h	DMSO	8.81 ± 7.3	4 h	DMSO	65.87 ± 0.15
4 h	Yoda1	199.99 ± 25.94	4 h	GSK	772 ± 84.21
4 h	PMA	264.13 ± 24.27	4 h	PMA	256.87 ± 86.01
E			F		
0.5 h	DMSO	9.49 ± 0.42	0.5 h	DMSO	9.5 ± 0.42
0.5 h	Yoda1	26.81 ± 1.82	0.5 h	GSK	22.71 ± 2.77
0.5 h	PMA	28 ± 2.48	0.5 h	PMA	28 ± 2.48
1 h	DMSO	11.5 ± 0.814	1 h	DMSO	11.5 ± 0.27
1 h	Yoda1	33.06 ± 0.77	1 h	GSK	25.63 ± 3.46
1 h	PMA	37.48 ± 3.8	1 h	PMA	37.48 ± 3.8
2 h	DMSO	13.34 ± 0.81	2 h	DMSO	13.34 ± 0.81
2 h	Yoda1	35.69 ± 5.7	2 h	GSK	36.25 ± 6.75
2 h	PMA	56.3 ± 9.15	2 h	PMA	56.3 ± 9.15
4 h	DMSO	19.07 ± 0.5	4 h	DMSO	19.07 ± 0.5
4 h	Yoda1	37.2 ± 1.12	4 h	GSK	26.19 ± 2.45
4 h	PMA	43.81 ± 17.09	4 h	PMA	43.81 ± 17.09

Suppl. table 2: ELISA mean raw values of Figure 2 in pg/ml.

**Figure 3: Ion channel-mediated JAM-A and TNFR1 release is specific for the corresponding mechanoresponsive ion channel and ADAM protease**					
**A**			**B**		
DMSO	DMSO	41.44 ± 6.31	DMSO	DMSO	46.88 ± 8.91
DMSO	GI	13.21 ± 2.38	DMSO	GI	27.48 ± 5.7
DMSO	GW	4.53 ± 2.61	DMSO	GW	17.01 ± 1.56
Yoda1	DMSO	241.89 ± 53.63	GSK	DMSO	434.07 ± 187.1
Yoda1	GI	107.71 ± 53.63	GSK	GI	251.56 ± 110.86
Yoda1	GW	16.58 ± 8.18	GSK	GW	22.44 ± 6.6
**C**			**D**		
DMSO	DMSO	10.56 ± 4.91	DMSO	DMSO	15.09 ± 4.4
DMSO	GI	3.09 ± 3.1	DMSO	GI	13.92 ± 5.7
DMSO	GW	0.84 ± 1.19	DMSO	GW	7.99 ± 6.66
Yoda1	DMSO	40.79 ± 6.09	GSK	DMSO	28.19 ± 7.84

Yoda1	GI	40.61 ± 4.2	GSK	GI	23.25 ± 4.68
Yoda1	GW	1.19 ± 1.68	GSK	GW	9.51 ± 6.54
	E			E	
DMSO	DMSO	21.15 ± 5.84	DMSO	DMSO	15.09 ± 4.4
DMSO	SalB	21.33 ± 4.44	DMSO	HC	16.59 ± 5.9
Yoda1	DMSO	100.71 ± 24.12	Yoda1	DMSO	28.19 ± 7.84
Yoda1	SalB	37.33 ± 17.15	Yoda1	HC	12.95 ± 4.88
	F			G	
DMSO	control	55.42 ± 36.1	DMSO	control	11.26 ± 0.98
DMSO	*PIEZO1*	65.56 ± 44.15	DMSO	*TRPV4*	10.76 ± 0.19
Yoda1	control	490.66 ± 113.57	Yoda1	control	226.24 ± 98.14
Yoda1	*PIEZO1*	160.65 ± 30.76	Yoda1	*TRPV4*	68.5 ± 62.67
PMA	control	960.07 ± 248.18	PMA	control	290.38 ± 49.06
PMA	*PIEZO1*	1065.04 ± 217.07	PMA	*TRPV4*	267.32 ± 45.66
	H			J	
DMSO	DMSO	42.28 ± 17.7	DMSO	DMSO	46.88 ± 8.91
DMSO	HC	38.73 ± 21.31	DMSO	SalB	44.22 ± 11.91
Yoda1	DMSO	267.75 ± 80.94	GSK	DMSO	434.07 ± 187.1
Yoda1	HC	280.24 ± 65.08	GSK	SalB	516 ± 254.13

Suppl. table 3: ELISA mean raw values of Figure 3 in pg/ml.

Figure 4: Piezo1 mediates the ADAM-dependent loss of VE-cadherin					
	C			D	
DMSO	DMSO	8321 ± 177580	DMSO	DMSO	9184 ± 1877.59
DMSO	GW	9825.67 ± 1703.03	DMSO	GW	11969 ± 1997.47
Yoda1	DMSO	6054.33 ± 1665.71	GSK	DMSO	5022.67 ± 1964.48
Yoda1	GW	7966 ± 760.63	GSK	GW	9120 ± 3208.84
	E			F	
DMSO	DMSO	28.10 ± 3.37	DMSO	DMSO	28.10 ± 3.37
DMSO	GW	28.15± 2.78	DMSO	GW	28.15± 2.78
Yoda1	DMSO	23.51 ± 2.97	GSK	DMSO	24.21 ± 3.01
Yoda1	GW	27.20 ± 2.88	GSK	GW	27.78 ± 2.35

Suppl. table 4: Flow cytometry mean raw values (C, D) and ELISA mean raw values in pg/ml (E, F) of Figure 4.

Suppl. Figure 4: Influence of Piezo1 activation on TNFR1 and JAM-A surface levels					
	A			B	
0.5 h	DMSO	390.6 ± 94.74	0.5 h	DMSO	31959.33 ± 20269.67
0.5 h	Yoda1	196.2 ± 42.78	0.5 h	Yoda1	23216.67 ± 15063.16
	C			D	
4 h	DMSO	183.67 ± 10.34	4 h	DMSO	2684.15 ± 900.54
4 h	Yoda1	212.33 ± 37.72	4 h	Yoda1	2611.84 ± 738.44
			4 h	PMA	1851.83 ± 374.79

Suppl. table 5: Flow cytometry mean raw values of Suppl. Fig. 4.

Suppl. Figure 5: Influence of Piezo1 and TRPV4 activation on ADAM10 and ADAM17 expression					
E			G		
0.5 h	DMSO	3884.33 ± 1986.50	0.5 h	DMSO	715.67 ± 142.46
0.5 h	Yoda1	3062.00 ± 1484.25	0.5 h	Yoda1	529.00 ± 101.26
0.5 h	GSK	3336.67 ± 1982.51	0.5 h	GSK	563.67 ± 38.72
1 h	DMSO	3019.00 ± 1945.30	1 h	DMSO	659.67 ± 185.63
1 h	Yoda1	3106.67 ± 2097.83	1 h	Yoda1	483.67 ± 121.56
1 h	GSK	3041.00 ± 2105.03	1 h	GSK	482.33 ± 74.55
2 h	DMSO	3602.00 ± 2071.75	2 h	DMSO	591.67 ± 152.90
2 h	Yoda1	3491.33 ± 2128.48	2 h	Yoda1	549.67 ± 15.11
2 h	GSK	3029.00 ± 2331.42	2 h	GSK	503.00 ± 47.69
4 h	DMSO	3370.67 ± 2322.04	4 h	DMSO	529.00 ± 83.26
4 h	Yoda1	3253.33 ± 2379.96	4 h	Yoda1	457.33 ± 68.16
4 h	GSK	3282.33 ± 2026.45	4 h	GSK	523.33 ± 25.42

Suppl. table 6: Flow cytometry mean raw values of Suppl. Fig. 5.

Suppl. Figure 6: Piezo1- and TRPV4-mediated ADAM activation is independent of PKC signaling					
A			B		
DMSO	DMSO	14.45 ± 12.09	DMSO	DMSO	39.34 ± 28.81
DMSO	BIM-2	12.12 ± 13.23	DMSO	BIM-2	41.67 ± 24.13
Yoda1	DMSO	104.13 ± 59.85	GSK	DMSO	260.1 ± 150.91
Yoda1	BIM-2	122.63 ± 82.7	GSK	BIM-2	270.59 ± 97.18
PMA	DMSO	180.07 ± 83.92	PMA	DMSO	680.33 ± 131.59
PMA	BIM-2	12.3 ± 12.47	PMA	BIM-2	107.05 ± 119.62
C					
DMSO	DMSO	4.05 ± 2.19			
DMSO	BIM-2	6.51 ± 3.1			
Yoda1	DMSO	36.52 ± 3.63			
Yoda1	BIM-2	39.59 ± 4.21			
GSK	DMSO	30.36 ± 2.76			
GSK	BIM-2	44.83 ± 9.33			
PMA	DMSO	87.23 ± 5.22			
PMA	BIM-2	8.92 ± 1.18			

Suppl. table 7: ELISA mean raw values of Suppl. Fig. 6 in pg/ml.

Suppl. Figure 7: Involvement of kinases in the Piezo1-mediated JAM-A release					
A			B		
DMSO	0 KN93	38.12 ± 1.86	DMSO	DMSO	165.57 ± 44.1
DMSO	0.3 µM KN93	49.42 ± 13.85	DMSO	U0126	103.25 ± 57.03
DMSO	1 µM KN93	43.28 ± 7.21	DMSO	SCH	113.76 ± 42.05
Yoda1	0 µM KN93	140.8 ± 81.59	Yoda1	DMSO	832.54 ± 459.52
Yoda1	0.3 µM KN93	108.82 ± 31.39	Yoda1	U0126	680.42 ± 443.52

Yoda1	1 µM KN93	189.5 ± 84.11	Yoda1	SCH	610.92 ± 355.48
C			D		
DMSO	0 µM LY	47.98 ± 0.96	DMSO	0 µM TAE	39.71 ± 5.47
DMSO	0.3 µM LY	47.34 ± 1.19	DMSO	0.3 µM TAE	45.85 ±10.53
DMSO	1 µM LY	47.03 ± 1.15	DMSO	1 µM TAE	42.54 ± 1.39
DMSO	3 µM LY	49.02 ± 2.45	DMSO	3 µM TAE	45.74 ± 14.55
DMSO	10 µM LY	51.66 ± 3.6	DMSO	10 µM TAE	40.69 ± 3.12
Yoda1	0 µM LY	111.9 ± 11.90	Yoda1	0 µM TAE	131.63 ± 42.79
Yoda1	0.3 µM LY	86.84 ± 8.4	Yoda1	0.3 µM TAE	106.07 ± 17.73
Yoda1	1 µM LY	94.14 ± 9.37	Yoda1	1 µM TAE	124.67 ± 31.73
Yoda1	3 µM LY	104.15 ± 4.53	Yoda1	3 µM TAE	140.96 ± 36.38
Yoda1	10 µM LY	118.29 ± 10.15	Yoda1	10 µM TAE	300.27 ± 106.39

Suppl. table 8: ELISA mean raw values of Suppl. Fig. 7 in pg/ml.

Target	Sequence (5'-3')	Temperature (°C)
hRPL13A_for	GCC CTA CGA CAA GAA AAG CG	60
hRPL13A_rev	TAC TTC CAG CCA ACC TCG TGA	
hJAM-A_for	TGA CAG AAC AAA GAA AGG GAC	60
hJAM-A_rev	GTG AAA CTA CAG ACA TCA GGG	
hHEY1_for	CCCAACTACATCTTCCCA	59
hHEY1_rev	GTCAAAGTAACCTTTCCCTC	
hCDH5_for	TCA AGC GTG AGT CCG CAA GAA	60
hCDH5_rev	AAT GAC AGC AGT GAG GTG GT	
hTNFR1_for	CAG TTC CAC CTT CAC CTC	59
hTNFR1_rev	GGG TCA TCA GTG TCT AGG	
hTRPV4_for	GTGACCTACATCATCCTCACCT	60
hTRPV4_rev	CCTCATCCACCCTGAAGCAC	
hPIEZO1_for	ATCTCAACCTCTTCCTCTTCC	57
hPIEZO1_rev	CGGGTATTTCTTCTCTGTCTC	
hADAM17_for	GAA GTG CCA GGA GGC GAT TA	57
hADAM17_rev	CGG GCA CTC ACT GCT ATT ACC	
hADAM10_for	GGA TTG TGG CTC ATT GGT GGG CA	61
hADAM10_rev	ACT CTC TCG GGG CCG CTG AC	
hPCNA_for	GAAGCACCAAACCAGGAG	59
hPCNA_rev	CACAGGAAATTACAACAGCA	
hMKi67_for	AGTTCCACAAATCCAACAC	59
hMKi67_rev	TCATCAGGGTCAGAAGAG	

Suppl. table 9: Primers used for qPCR with corresponding sequences and annealing temperatures.
